# Study on Axial Compression Performance of CFRP-Aluminum Alloy Laminated Short Tubes

**DOI:** 10.3390/ma18153480

**Published:** 2025-07-24

**Authors:** Xiaoqun Luo, Yanheng Li, Li Wang, Xiaonong Guo

**Affiliations:** 1 College of Civil Engineering, Tongji University, Shanghai 200092, China; luoxiaoqun@tongji.edu.cn (X.L.); 2330671@tongji.edu.cn (Y.L.); 2 Shanghai Construction No.1 (Group) Co., Ltd., Shanghai 200120, China; gcyjy@sc1gc.com.cn

**Keywords:** CFRP-AL tubes, axial compression performance, small slenderness ratio, strength failure

## Abstract

CFRP possesses the advantages of lightweight and high strength, but its cost is relatively high, and its ductility is insufficient; aluminum alloys have a relatively low cost and good ductility. This paper develops a CFRP-aluminum alloy laminated tube (CFRP-AL tube), which combines the advantages of CFRP and aluminum alloy. Such composite components have broad application prospects in the field of spatial structures. The CFRP-AL tubes were studied by experimental, numerical, and theoretical research on their axial compression performance in this paper. Firstly, the standard tensile test was carried out on 6061-T6 aluminum alloy. Combining the test results and references, the Johnson–Cook hardening model parameters of aluminum alloy were determined. The tensile test of CFRP was conducted to determine its material parameters. Based on composite material mechanics and fracture mechanics, a composite progressive damage model for the CFRP-AL tube was established. Secondly, axial compression tests were carried out on 27 CFRP-AL tubes and 3 aluminum alloy tubes with a small slenderness ratio. The test results show that the typical failure mode of CFRP-AL tubes with small slenderness ratios is strength failure, and the ultimate bearing capacity rises by 11~31% compared to aluminum alloy tubes. Thirdly, a user material subroutine capable of simulating CFRP failure was developed. Based on the user material subroutine, the effect of the initial imperfection, the fiber layer angle, the fiber layer thickness, the slenderness ratio, the diameter-thickness ratio and the CFRP volume ratio were discussed. And the failure mechanism and response of the CFRP-AL tubes under the axial compression were obtained. Finally, based on the strength theory, the formula predicting the bearing capacity of the strength failure was established, and the results of the formula were in a good agreement with the experimental and numerical results.

## 1. Introduction

Fiber reinforced plastics (FRP) are high-performance materials composed of reinforcing fibers and epoxy resin in specific proportions. Carbon fiber reinforced plastics (CFRP) exhibit high specific strength, specific stiffness, and high energy absorption, making them widely used in aerospace and automotive manufacturing.

FRP/metal composite members combine lightweight, high-strength FRP with ductile metal materials. A considerable amount of research has been conducted on the interaction between CFRP and steel or concrete [1,2,3]. Compared to single materials, these composite components significantly enhance stiffness and strength while maintaining good ductility and greatly improve energy absorption capacity. Such composite components can reduce the weight of spatial structures while meeting energy absorption requirements in structural applications.

In fact, aluminum alloy and FRP composite components have been successfully applied in fields such as aerospace, vehicles, and ships [4,5]. However, in civil engineering, there have been only a few application studies [6,7], most of which focus primarily on theoretical research [8,9,10]. In addition, most existing studies on FRP/metal tubes focus on the combination method of FRP wrapping around metal tubes, such as CFRP/GFRP/AFRP/BFRP wrapping steel/aluminum alloy square or circular tubes. Only limited research has addressed the layered composite form of metal/FRP/metal tubes. In 2017, Zhu et al. [11] explored the quasi-static axial crushing energy absorption characteristics of aluminum alloy/CFRP/aluminum alloy tubes. Their study found that the total energy absorption and peak load of the composite tubes were higher than the sum of individual components. In 2020, Han et al. [12] optimized the bonding performance between aluminum alloy and CFRP using spinning technology and studied the quasi-static axial crushing energy absorption characteristics. In 2022, Mansor et al. [13] investigated the energy absorption performance of aluminum alloy/GFRP composite circular tubes in 2/1 and 3/2 layered forms under axial low-speed impact, concluding that it is an excellent energy-absorbing composite component.

In the early days, FRP materials were mostly used for reinforcement and seismic resistance in the field of civil engineering. In the 1960s, Germany used GFRP plates in bridge reinforcement. In the 1980s, after the Hanshin earthquake, Japan widely used CFRP to repair damaged reinforced concrete columns [14]. At the end of the last century, FRP reinforcement technology for concrete was effectively applied in civil engineering. Regarding the performance of composite materials, Ronagh et al. [15] studied the seismic performance of reinforced concrete structures strengthened with FRP composites, confirming that the lateral bearing capacity is significantly increased when using the two composites. The improvement effect of CFRP is twice that of GFRP. Kavitha et al. [16] compared the ultimate bearing capacity of concrete specimens wrapped with CFRP and GFRP, respectively, under axial compressive load. The results show that the CFRP specimens have higher ultimate bearing capacity and better energy absorption capacity. In studies on the mechanical properties of FRP composites, many scholars have adopted progressive damage models for finite element simulations. For example, Ding et al. [17] developed a progressive damage model based on ABAQUS/VUMAT to investigate the bending behavior of CFRP box girders; Shen et al. [18] constructed a progressive damage model for CFRP laminates to predict failure modes.

Due to the excellent properties of FRP, such as high strength and high modulus, FRP/metal composite components have attracted widespread attention from researchers. Current research focuses on the static performance of components, such as axial compression and bending, using methods including experimental, numerical, and theoretical research. Regarding the axial compression performance of FRP/metal composite components, Zhao et al. [19] reviewed previous studies on the axial compression performance of FRP/steel composite components. Ritchie et al. [20] conducted axial compression stability tests on slender ultra-high modulus CFRP/H-shaped steel composite components and found that the enhancement effect on the stability bearing capacity of the composite components increased with the modulus of CFRP, initial bending, and CFRP reinforcement ratio. Shaat et al. [21,22] performed axial compression tests on CFRP/square steel tubes with different slenderness ratios. The results showed that, compared to steel tubes, the ultimate bearing capacity of composite short tubes increased by 18%, while that of composite long tubes increased by 13% to 23%. Kumar et al. [23,24] studied the mechanical properties of slender CFRP/circular steel tube composite components under axial static loads and axial cyclic loads. The results indicated that the bearing capacity of composite components was significantly improved under both static and cyclic loads. Chen et al. [25] found that CFRP could enhance the local buckling performance of square steel tubes. Feng et al. [26] conducted axial compression tests on short CFRP/aluminum alloy square tube and CFRP/aluminum alloy circular tube composite components, analyzing the effects of CFRP layer count, diameter-to-thickness ratio, width-to-thickness ratio, and fiber orientation, and studied their elastoplastic stability. Ye Lieping et al. [27,28] performed axial compression stability tests on slender CFRP/aluminum alloy circular tube composite components and established a composite shell structure model. They found that compared to pure aluminum alloy shell structures, the composite shell structures exhibited higher ultimate bearing capacity and lower sensitivity to initial defects. Shin et al. [29] studied GFRP/aluminum alloy square tube composite components, demonstrating that the composite components possess superior axial compression and bending performance. Gao et al. [30] conducted axial compression tests on slender CFRP/circular steel tubes, revealing that CFRP enhanced the axial strength and stiffness of the components, with increases of 28~124% and 25~105%, respectively.

In summary, FRP/metal composite components exhibit lightweight and high-strength characteristics under static loads. However, most existing studies adopt the form of FRP externally bonded to metal components, with few focusing on the combination method where FRP is sandwiched between metal layers. Preliminary research indicates that this type of component possesses superior performance [11,12,13], making it necessary to conduct further studies on its static performance.

This study investigates the axial compression performance of CFRP-AL tubes by a combination of experimental tests and finite element analysis. Firstly, axial compression tests were conducted considering factors such as the fiber winding angle and fiber thickness, analyzing the failure modes and ultimate bearing capacity of the components. Secondly, using the progressive damage theory, a finite element model for the axial compression of composite components was established using a user material subroutine, and the model’s validity was verified. Subsequently, parametric analysis of the axial compression performance of the composite components was carried out based on the finite element model, revealing the failure mechanisms and destruction patterns of the composite components. Finally, calculation formulas were proposed for the ultimate bearing capacity of the composite components under strength failure.

## 2. Material Properties and Specimen Fabrication

Material property tests were first conducted separately for aluminum alloy and CFRP to obtain the material parameters under quasi-static conditions. Meanwhile, an appropriate dynamic constitutive model for aluminum alloy was selected, and failure initiation criteria and damage evolution rules suitable for various failure modes—including CFRP fiber tensile failure, fiber compressive failure, resin tensile failure, and resin compressive failure—were established. A material constitutive model user subroutine (VUMAT) based on the ABAQUS/Explicit (6.14) module was also developed.

### 2.1. Constitutive Model of Aluminum Alloy

#### 2.1.1. Material Property Test

The CFRP-AL tubes are made of carbon fiber, epoxy resin, and aluminum alloy. Both the inner and outer aluminum alloy tubes are made of 6061-T6 aluminum alloy, while the intermediate CFRP layer consists of T700 carbon fiber reinforced E51 epoxy resin.

**Figure 1:**
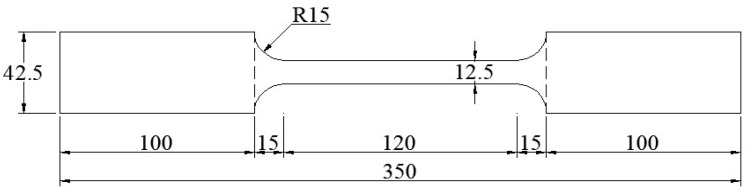
Aluminum alloy specimen size for testing.

To determine the mechanical properties of the 6061-T6 aluminum alloy circular tubes, standard monotonic tensile tests were conducted. For the two thicknesses of aluminum alloy tubes used in the experiment, specimens were sampled from circular tubes with cross-sectional design dimensions of 3.0 mm and 1.5 mm thickness, respectively. The samples were taken longitudinally along the tube length. Four samples were taken for each thickness, resulting in a total of eight specimens. Specific dimensions are shown in Figure 1.

The loading method was performed according to the provisions of the national standard GB/T 228.1-2010 “Metallic Materials—Tensile Testing—Part 1: Method of Test at Room Temperature.” [31] The specimens were uniformly stretched to fracture at a loading rate of 1 mm/min.

**Figure 2:**
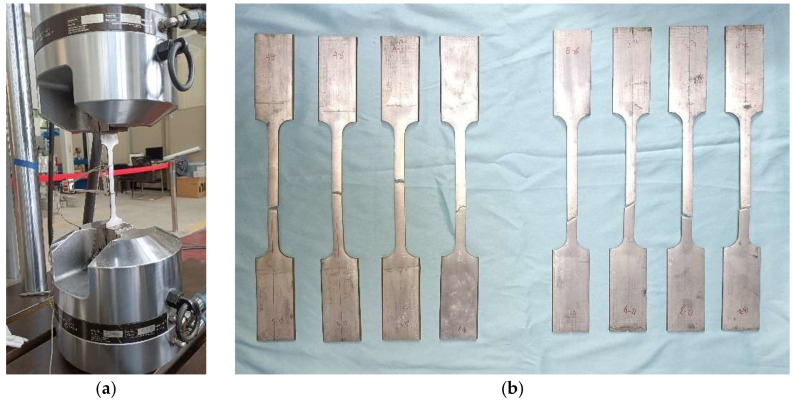
Tensile testing of aluminum alloy specimens. (**a**) Photos of the test device; (**b**) Photos of the damaged specimens.

**Figure 3:**
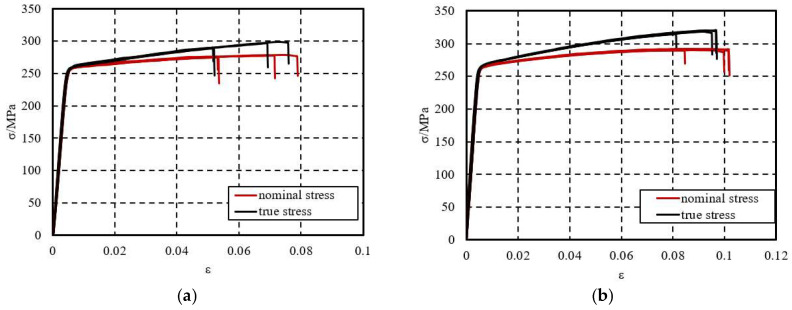
Stress–strain curve of 6061-T6 aluminum alloy tensile specimen. (**a**) 1.5 mm thickness aluminum alloy; (**b**) 3.0 mm thickness aluminum alloy.

The testing process and photos of the fractured specimens are shown in Figure 2. During the test, significant radial contraction may occur in the gauge length section, and directly using the nominal stress–strain relationship may lead to errors. Therefore, it is necessary to convert the nominal stress and nominal strain data obtained from the test into true stress and true strain. The curves of true stress-true strain and nominal stress-nominal strain for the specimens are shown in Figure 3.

From the uniaxial tensile test, mechanical property parameters such as elastic modulus, yield strength, and ultimate tensile strength can be obtained. Based on the test results, the average values of the measured results for the eight specimens are taken as the mechanical property indicators of the 6061-T6 aluminum alloy in this study, as shown in Table 1 and Table 2.

#### 2.1.2. Failure Criteria and Damage Evolution Criteria

Under low-speed impact loading, the strain rate effect of aluminum alloy materials needs to be considered. In this study, the Johnson–Cook hardening and damage model (J–C) [32] is adopted. The expression of the J–C model is shown in Equation (1), and the specific form of the J–C fracture criterion is presented in Equation (2):

(1) $$\sigma_{\mathrm{eq}}=(A+B{\varepsilon_{\mathrm{eq}}}^{n})(1+C\ln{\dot{\varepsilon }}^{*})(1-T^{*m})$$

(2) $$\varepsilon_{f}=[D_{1}+D_{2}\exp(D_{3}\sigma^{*})](1+D_{4}\ln{\dot{\varepsilon }}^{*})(1+D_{5}T^{*})$$

*A*, *B*, *C*, *m*, *n*—Material-related constants;
$\sigma_{\mathrm{eq}}$—Equivalent stress;
$\varepsilon_{\mathrm{eq}}$—Equivalent plastic strain;
${\dot{\varepsilon }}^{*}$—Dimensionless equivalent plastic strain rate;
$T^{*}$—Dimensionless temperature;
*D*_1_~*D*_5_—Material performance parameters;
$\sigma^{*}$—Stress triaxiality;

Where ${\dot{\varepsilon }}^{*}=\dot{\varepsilon }/{\dot{\varepsilon }}_{0}$, $\dot{\varepsilon }$ is the current strain rate, ${\dot{\varepsilon }}_{0}$ is the reference strain rate, $T^{*m}=(T-T_{r})(T_{m}-T_{r})$, *T* is the current temperature of the material, $T_{m}$ is the melting temperature of the material, $T_{r}$ is the reference temperature, which is generally taken as room temperature.

$\sigma^{*}$ is defined as the ratio of hydrostatic pressure to equivalent stress, $\sigma^{*}=\sigma_{m}/\sigma_{\mathrm{eq}}=(\sigma_{1}+\sigma_{2}+\sigma_{3})/(3\sigma_{\mathrm{eq}})$, where $\sigma_{m}$ is the hydrostatic pressure, and $\sigma_{1}\sim \sigma_{3}$ are the first, second, and third principal stresses, respectively.

Schwer et al. [33] calibrated the J–C fracture model parameters of 6061-T6 aluminum alloy through experiments and finite element analysis. Meanwhile, our research team [34] conducted material property tests on various aluminum alloys and calibrated the fracture toughness parameters of the VGM and SMCS models for the 6061-T6 aluminum alloy. Based on this, the J–C model parameters D1 and D2 were fitted, while D3 to D5 were adopted from the results in reference [33]. Therefore, combining the monotonic tensile loading test results of aluminum alloy in this study, we obtained the J–C hardening model parameters shown in Table 3 and the J–C fracture criterion parameters shown in Table 4.

**Figure 4:**
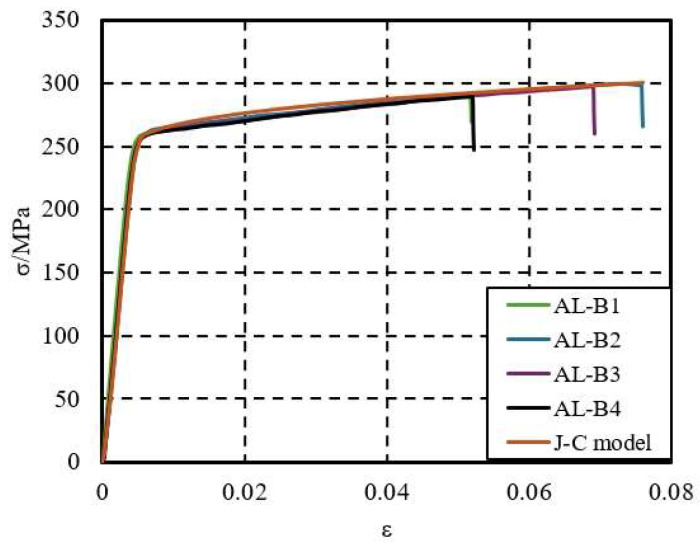
Comparison of experimental true stress–strain curves with the J–C model.

**Figure 5:**
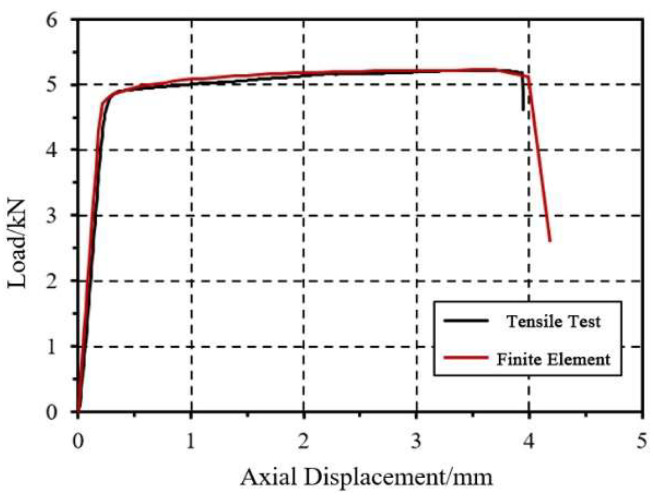
Comparison of load–displacement curves for tensile specimens.

**Figure 6:**
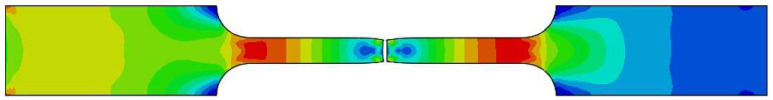
Finite Element Model Failure Mode.

To verify the rationality of the aluminum alloy constitutive model, a comparison was made between the true stress–strain curve obtained from aluminum alloy experiments and the stress–strain curve derived from the Johnson–Cook model used in this study, as shown in Figure 4. The two curves show good agreement. Additionally, finite element models of the aluminum alloy monotonic tensile specimen AL-B2 were established. The load–displacement curve from the experiment was compared with the finite element results, as shown in Figure 5. The failure mode of the specimen, depicted in Figure 6, also matches well with the experimental results.

### 2.2. Constitutive Model of CFRP

#### 2.2.1. Material Properties Test

**Figure 7:**
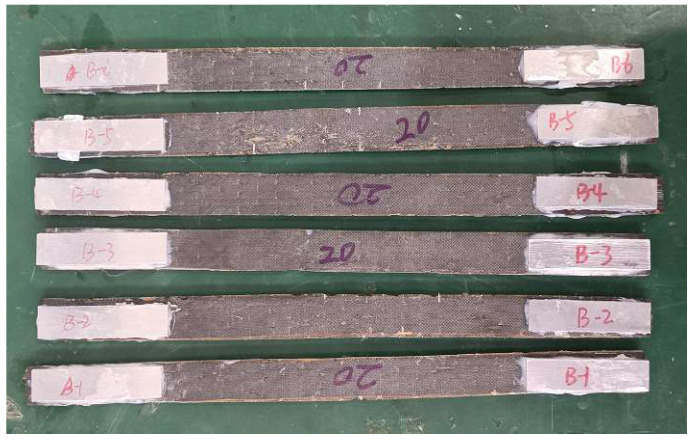
CFRP material specimens.

**Figure 8:**
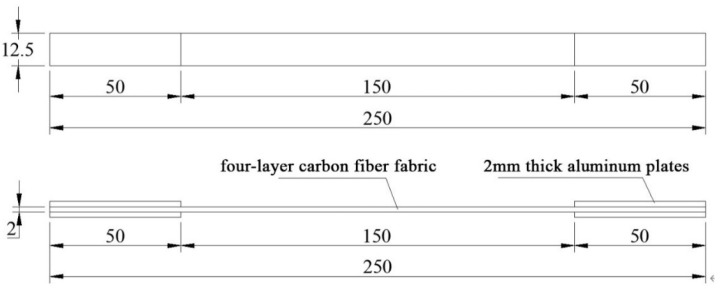
The dimensions of CFRP material specimens.

**Figure 9:**
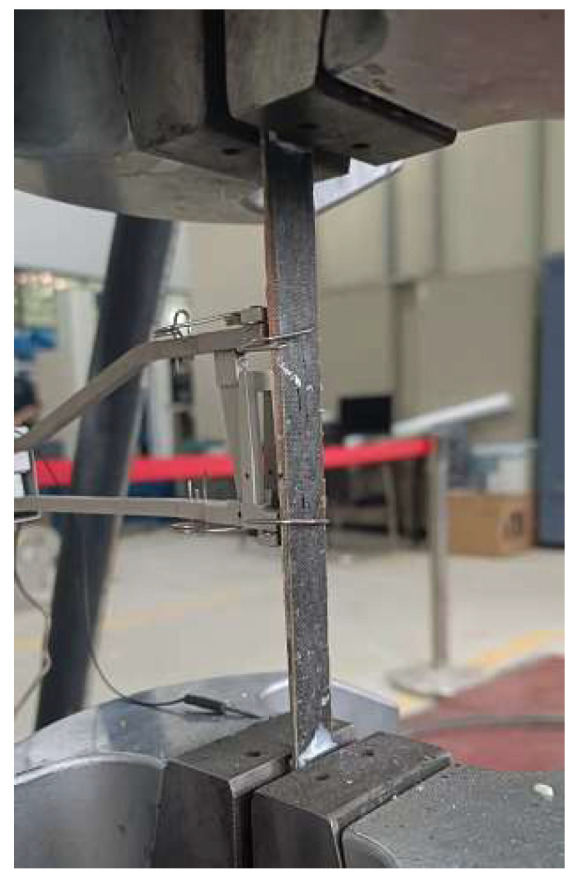
Loading device of CFRP material specimens.

CFRP has the characteristic of orthotropy. In this study, Toray T700 (300 g/m^2^) carbon fiber provided by Toray Carbon Fiber (Guangdong) Co., Ltd., (Foshan City, China) was used. Tensile mechanical tests were conducted on the carbon fiber fabric, and other mechanical properties were calculated based on formulas from the literature. According to GB/T 3354-2014 “Test method for tensile properties of orientation fiber reinforced polymer matrix composite materials“ [35], six CFRP longitudinal tensile specimens were designed. Aluminum alloy plates were used as reinforcement pieces at both ends of the specimens, bonded with structural adhesive for easy gripping, as shown in Figure 7. The dimensions of the specimens are shown in Figure 8, and the loading device is shown in Figure 9. The specimens were aligned with the central axis of the fixture and were pulled to failure at a uniform loading rate of 1 mm/min.

For T700 carbon fiber, *M_u_* = 300 g/m^2^ and *ρ_c_* = 1.8 g/cm^3^. The results are shown in Table 5.

The material properties data of the epoxy resin used in the test were obtained from the quality inspection report provided by the manufacturer, as shown in Table 6.

#### 2.2.2. CFRP Engineering Constants

The engineering constants of CFRP are calculated using the commonly used Halpin–Tsai formula, as shown in the following formulas.

(3) $$E_{1}=E_{f}V_{f}+E_{m}V_{m}$$

(4) $$v_{12}=v_{f}V_{f}+v_{m}V_{m}$$

(5) $$\frac{M}{M_{m}}=\frac{1+\xi \eta V_{f}}{1-\eta V_{f}}$$

(6) $$\eta =\frac{(M_{f}/M_{m})-1}{(M_{f}/M_{m})+\xi }$$

(7) $$X_{1}=X_{f}V_{f}+X_{m}V_{m}$$

*E*_1_—Elastic Modulus in the 1 Direction of Composite Material;
*V*_f_—Fiber Volume Fraction;
*V*_m_—Matrix Volume Fraction;
*M*—Composite material moduli, such as *E*_2_, *G*_12_, *v*_23_;
*M*_f_—Fiber moduli, such as *E*_f_, *G*_f_, *v*_f_;
*M*_m_—Matrix moduli, such as *E*_m_, *G*_m_, *v*_m_;
*X*_1_—Strength in the 1 Direction of Composite Material
*X*_f_—Fiber Strength;
*X*_m_—Matrix Strength.

In the formula, *ξ* represents the degree of reinforcement of the fiber-reinforced material. The Poisson’s ratio of the epoxy resin used in the test is *v*_m_ = 0.37; the Poisson’s ratio of carbon fiber is *v*_f_ = 0.37. According to the literature [36], *V*_f_ and *V*_m_ are taken as 0.33 and 0.67, respectively. $\xi_{E_{2}}=2$, $\xi_{G_{12}}=1$, and $\xi_{v_{12}}=1.2$.

The shear modulus is calculated using the following formula.

(8) $$G=\frac{E}{2(1+v)}$$

The calculated engineering constants of CFRP are shown in Table 7.

In this context, *E*, *v*, and *G* represent the elastic modulus, Poisson’s ratio, and shear modulus, respectively. By combining the strengths of carbon fiber and epoxy resin from Table 5 and Table 6, and using Equation (7), the directional strengths and fracture energy of CFRP are calculated. According to the literature [37], the results for the directional strengths and fracture energy of CFRP are shown in Table 8.

#### 2.2.3. Failure Criteria and Damage Evolution Criteria

The possible failure modes of CFRP-AL tubes can be divided into two categories: one is intralaminar failure, including fiber tensile failure, fiber compressive failure, matrix tensile failure, and matrix compressive failure; the second is interlaminar failure. This paper uses the Hashin failure criteria [38] to predict fiber tensile failure, fiber compressive failure, and matrix tensile failure, and the Puck failure criteria to predict matrix compressive failure [37,39]. The specific failure criteria are as follows.

(1) Fiber Tensile Failure Criterion ($\sigma_{11}\geq 0$):

(9) $$F_{1}^{t}={\left(\frac{\sigma_{11}}{X_{T}}\right)}^{2}+{\left(\frac{\sigma_{12}}{S_{12}}\right)}^{2}+{\left(\frac{\sigma_{13}}{S_{13}}\right)}^{2}\geq 1$$

(2) Fiber Compressive Failure Criterion ($\sigma_{11}<0$):

(10) $$F_{1}^{c}={\left(\frac{\sigma_{11}}{X_{C}}\right)}^{2}\geq 1$$

(3) Matrix Tensile Failure Criterion ($\sigma_{22}\geq 0$):

(11) $$F_{2}^{t}={\left(\frac{\sigma_{22}}{Y_{T}}\right)}^{2}\geq 1$$

(4) Matrix Compressive Failure Criterion ($\sigma_{22}<0$):

(12) $$F_{2}^{c}={\left(\frac{\tau_{\mathrm{nt}}(\theta )}{S_{23}^{A}+\mu_{\mathrm{nt}}\sigma_{n}(\theta )}\right)}^{2}+{\left(\frac{\tau_{\mathrm{nl}}(\theta )}{S_{12}+\mu_{\mathrm{nl}}\sigma_{n}(\theta )}\right)}^{2}\geq 1$$

*X*, *Y*, *Z*, *S*—Material strength, where the subscript T denotes tension and C denotes compression;
$\sigma_{n}(\theta )$, $\tau_{\mathrm{nl}}(\theta )$, $\tau_{\mathrm{nt}}(\theta )$—Stress components on the fracture surface;
θ—The angle between the fracture surface and the cross-section;
$\mu_{\mathrm{nt}}$, $\mu_{\mathrm{nl}}$—Friction coefficient based on the Mohr–Coulomb failure theory;
$S_{23}^{A}$—Shear strength on the fracture surface;
ϕ—Material friction angle;
$\sigma_{ij}$—Directional stress, where i = j denotes normal stress, and i ≠ j denotes in-plane shear stress.

In the progressive failure process of composite materials, damage initiates when the stress state of an element reaches the failure criterion. At this point, it is necessary to establish a damage evolution model to degrade the material properties. This paper adopts a linear degradation model based on fracture toughness to simulate the failure of CFRP-AL composite material, where the functional form of the damage state variable d is as follows [40].

(1) Fiber Tensile Damage:

(13) $$d_{f}^{t}=\frac{\varepsilon_{1}^{\mathrm{ft}}}{\varepsilon_{1}^{\mathrm{ft}}-\varepsilon_{1}^{0t}}\left(1-\frac{\varepsilon_{1}^{0t}}{\varepsilon_{1}}\right)$$

(2) Fiber Compressive Damage:

(14) $$d_{f}^{c}=\frac{\varepsilon_{1}^{\mathrm{fc}}}{\varepsilon_{1}^{\mathrm{fc}}-\varepsilon_{1}^{0c}}\left(1-\frac{\varepsilon_{1}^{0c}}{\varepsilon_{1}}\right)$$

(3) Matrix Tensile Damage:

(15) $$d_{m}^{t}=\frac{\varepsilon_{2}^{\mathrm{ft}}}{\varepsilon_{2}^{\mathrm{ft}}-\varepsilon_{2}^{0t}}\left(1-\frac{\varepsilon_{2}^{0t}}{\varepsilon_{2}}\right)$$

(4) Matrix Compressive Damage:

(16) $$d_{m}^{c}=\frac{\gamma_{r}^{\mathrm{fc}}}{\gamma_{r}^{\mathrm{fc}}-\gamma_{r}^{0c}}\left(1-\frac{\gamma_{r}^{0c}}{\gamma_{r}}\right)\;$$

(17) $$\gamma_{r}=\sqrt{\gamma_{\mathrm{nt}}^{2}+\gamma_{\mathrm{nl}}^{2}}$$

(18) $$\gamma_{\mathrm{nt}}=-\varepsilon_{22}\sin\theta \cos\theta +\varepsilon_{33}\sin\theta \cos\theta +\gamma_{23}(\cos^{2}\theta -\sin^{2}\theta )$$

(19) $$\gamma_{\mathrm{nl}}=\gamma_{12}\cos\theta +\gamma_{13}\sin\theta$$

(20) $$\gamma_{r}^{f}=\frac{2G_{2C}^{c}}{S_{r}^{0}L^{c}}$$

(21) $$S_{r}^{0}=\sqrt{{\left(\tau_{\mathrm{nt}}^{0}(\theta )\right)}^{2}+{\left(\tau_{\mathrm{nl}}^{0}(\theta )\right)}^{2}}$$

(22) $$\tau_{\mathrm{nt}}^{0}(\theta )=-\sigma_{22}\sin\theta \cos\theta +\sigma_{33}\sin\theta \cos\theta +\tau_{23}(\cos^{2}\theta -\sin^{2}\theta )$$

(23) $$\tau_{\mathrm{nl}}^{0}(\theta )=\tau_{31}\sin\theta +\tau_{21}\cos\theta$$

In which *t* and *c* denote the tensile and compressive states, respectively, $d_{f}^{t}$ and $d_{f}^{c}$ represent the fiber tensile damage state variable and fiber compressive damage state variable, respectively, while $d_{m}^{t}$ and $d_{m}^{c}$ represent the matrix tensile damage state variable and matrix compressive damage state variable, respectively. $\varepsilon_{i}$ is the actual strain value of the element in the *i* direction (*i* = 1, 2), and $\varepsilon_{i}^{0t}$ and $\varepsilon_{i}^{\mathrm{ft}}$ are the strain values at the initial failure moment and the final failure moment of the material, respectively.

(24) $$\begin{array}{l} \varepsilon_{i}^{0t}=\frac{\sigma_{i}^{t/c}}{C_{ii}}\\ \varepsilon_{i}^{\mathrm{ft}}=\frac{2G_{iC}^{t/c}}{\sigma_{i}^{t/c}L^{c}} \end{array}$$

where $\sigma_{i}^{t/c}$ is the tensile/compressive strength of the element in the *i* direction. $C_{ii}$ is the stiffness matrix element. $G_{iC}^{t/c}$ is the tensile/compressive fracture toughness value of the element in the *i* direction. $L_{c}$ is the characteristic length of the element.

$\gamma_{r}$ is the shear strain on the fracture surface, $\gamma_{r}^{0c}$ is the damage initiation shear strain on the fracture surface, $\gamma_{r}^{\mathrm{fc}}$ is the final failure shear strain on the fracture surface, and $\tau_{\mathrm{nt}}^{0}(\theta )$ and $\tau_{\mathrm{nl}}^{0}(\theta )$ are the shear stresses in two directions at the damage initiation on the fracture surface.

The damage stiffness of CFRP considering damage is as follows [41]:

(25) $$\begin{array}{l} C_{11}^{d}=(1-d_{f})C_{11}\\ C_{22}^{d}=(1-d_{f})(1-d_{m})C_{22}\\ C_{33}^{d}=(1-d_{f})(1-d_{m})C_{33}\\ C_{12}^{d}=(1-d_{f})(1-d_{m})C_{12}\\ C_{23}^{d}=(1-d_{f})(1-d_{m})C_{23}\\ C_{13}^{d}=(1-d_{f})(1-d_{m})C_{13}\\ C_{44}^{d}=(1-d_{f})(1-s_{\mathrm{mt}}d_{m}^{t})(1-s_{\mathrm{mc}}d_{m}^{c})C_{44}\\ C_{55}^{d}=(1-d_{f})(1-s_{\mathrm{mt}}d_{m}^{t})(1-s_{\mathrm{mc}}d_{m}^{c})C_{55}\\ C_{66}^{d}=(1-d_{f})(1-s_{\mathrm{mt}}d_{m}^{t})(1-s_{\mathrm{mc}}d_{m}^{c})C_{66} \end{array}$$

where $d_{f}$ is the damage variable related to fiber damage, and $d_{m}$ is the damage variable related to matrix damage, calculated by the following formula:

(26) $$d_{f}=1-(1-d_{f}^{t})(1-d_{f}^{c})$$

(27) $$d_{m}=1-(1-d_{m}^{t})(1-d_{m}^{c})$$

$s_{\mathrm{mt}}$ represents the influence factor of matrix tension on the loss of matrix shear stiffness, and $s_{mc}$ represents the influence factor of matrix compression on the loss of matrix shear stiffness. In this paper, they are set as $s_{\mathrm{mt}}=0.9$ and $s_{\mathrm{mc}}=0.5$.

### 2.3. Interface Constitutive Model

The CFRP-AL tubes are composed of CFRP and aluminum alloy layers. The interfaces between CFRP layers and between CFRP and aluminum alloy layers are bonded with epoxy resin. The interlaminar stress is mainly transmitted through the interface layer, making interlayer interface failure likely under load. Therefore, it is crucial to determine a reasonable interlayer interface constitutive model. Existing studies show that the Cohesive Zone Model can effectively simulate the initiation and propagation of adhesive cracks.

**Figure 10:**
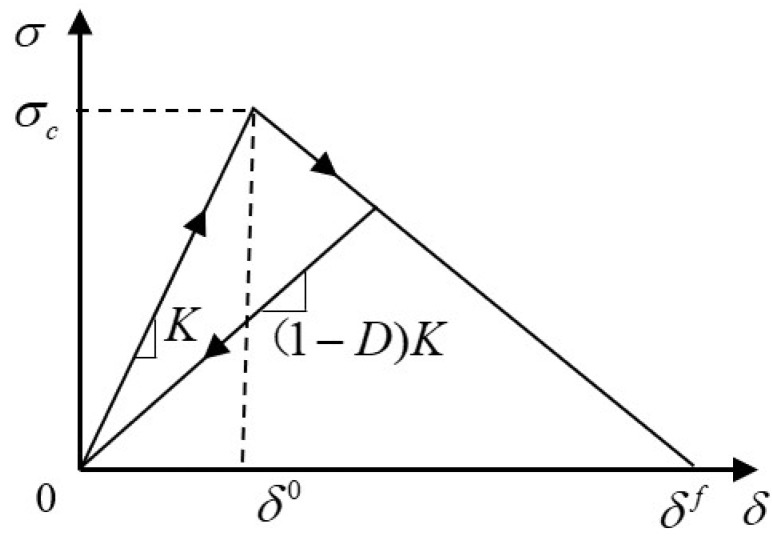
Bilinear slip model.

Due to its simplicity, the bilinear slip model is widely used in ABAQUS. As shown in Figure 10, the stresses acting on the interface are $\sigma_{n}$, $\sigma_{s}$, $\sigma_{t}$, and the opening displacements are $\delta_{n}$, $\delta_{s}$, $\delta_{t}$. The constitutive relationship is as follows:

(28) $$\left\{\begin{array}{c} \sigma_{n}\\ \sigma_{s}\\ \sigma_{t} \end{array}\right\}=\left[\begin{array}{ccc} K_{\mathrm{nn}} & K_{\mathrm{ns}} & K_{\mathrm{nt}}\\ K_{\mathrm{sn}} & K_{\mathrm{ss}} & K_{\mathrm{st}}\\ K_{\mathrm{tn}} & K_{\mathrm{ts}} & K_{\mathrm{tt}} \end{array}\right]\left\{\begin{array}{c} \delta_{n}\\ \delta_{s}\\ \delta_{t} \end{array}\right\}$$

For the interface region, the normal stress is not significantly related to the stresses in the other two directions. Therefore, the coupling terms can be set to zero, and the decoupled constitutive relationship is as follows:

(29) $$\left\{\begin{array}{c} \sigma_{n}\\ \sigma_{s}\\ \sigma_{t} \end{array}\right\}=\left[\begin{array}{ccc} K_{\mathrm{nn}} & 0 & 0\\ 0 & K_{\mathrm{ss}} & 0\\ 0 & 0 & K_{\mathrm{tt}} \end{array}\right]\left\{\begin{array}{c} \delta_{n}\\ \delta_{s}\\ \delta_{t} \end{array}\right\}$$

Additionally, it is necessary to define interface damage initiation and damage evolution to determine the state of the element. When the stress or strain reaches the failure criterion, the material begins to degrade until the fracture energy or displacement reaches a critical value, at which point the material completely fails. This paper uses the quadratic stress criterion (QUADSCRT) to consider the initiation of failure under complex stress conditions in the adhesive layer. After reaching the damage initiation,

(30) $$\left\{\begin{array}{c} \sigma_{n}\\ \sigma_{s}\\ \sigma_{t} \end{array}\right\}=\left[\begin{array}{ccc} (1-D)K_{\mathrm{nn}} & 0 & 0\\ 0 & (1-D)K_{\mathrm{ss}} & 0\\ 0 & 0 & (1-D)K_{\mathrm{tt}} \end{array}\right]\left\{\begin{array}{c} \delta_{n}\\ \delta_{s}\\ \delta_{t} \end{array}\right\}$$

The energy-based mixed-mode damage evolution model in ABAQUS and the B-K criterion are used to consider interface mixed failure. Referring to Reference [42], the interlayer interface constitutive parameters are shown in Table 9:

## 3. Axial Compression Test of Short CFRP-AL Tubes

### 3.1. Test Plan

#### 3.1.1. Specimen Fabrication

**Figure 11:**
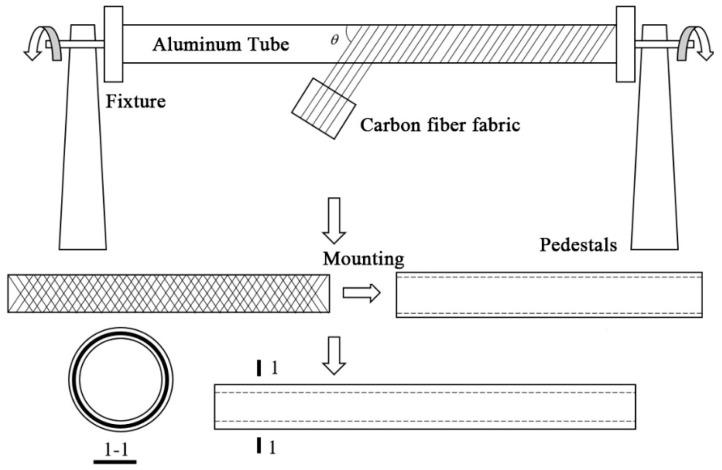
Schematic Diagram of the Composite Component Fabrication Process.

The manufacturing process of CFRP-AL tubes is similar to that of CFRP tubes. The specimens in this paper are produced using the hand lay-up process. The fabrication process is shown in Figure 11. The specific steps are as follows:

1. Surface Treatment: The aluminum alloy tubes used are 6061-T6 aluminum alloy tubes. The prepared aluminum alloy tubes are cut to the required length. To ensure a certain bonding strength, the metal surface must undergo some surface treatment before bonding [43,44]. Common surface treatments include chemical solvent cleaning and mechanical treatment. For simplicity, acetone solution cleaning is chosen for surface treatment in this study. The outer surface of the inner aluminum alloy tube and the inner surface of the outer aluminum alloy tube are sanded with sandpaper and cleaned with acetone.
2. Carbon Fiber Fabric Winding and Epoxy Resin Application: Carbon fiber fabric is manually wound layer by layer onto the outer surface of the inner aluminum alloy tube, followed by the application of epoxy resin. The assembly is slightly heated to enhance the flowability of the epoxy resin.
3. Assembly: While the epoxy resin has thorough wettability and good flowability, the carbon fiber-wrapped inner aluminum alloy tube is rotated and assembled into the outer aluminum alloy tube along the fiber angle. During the rotational assembly, the outer aluminum alloy tube exerts some pressure on the inner tube, causing the carbon fiber fabric to tighten and extrude excess epoxy resin, thereby reducing bubbles in the composite material and increasing the fiber volume content.
4. Curing and Molding: The assembled composite tube is left to cure naturally at room temperature for 7 days.
5. Post-Processing: After curing, the composite tube product is removed and cut to the required dimensions for testing. The edges and burrs of the specimens are polished.

**Figure 12:**
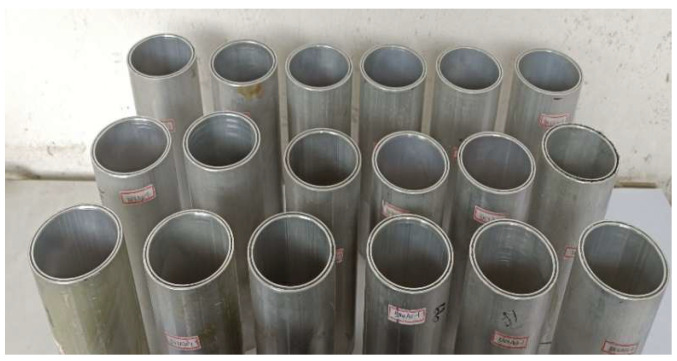
Actual Picture.

**Figure 13:**
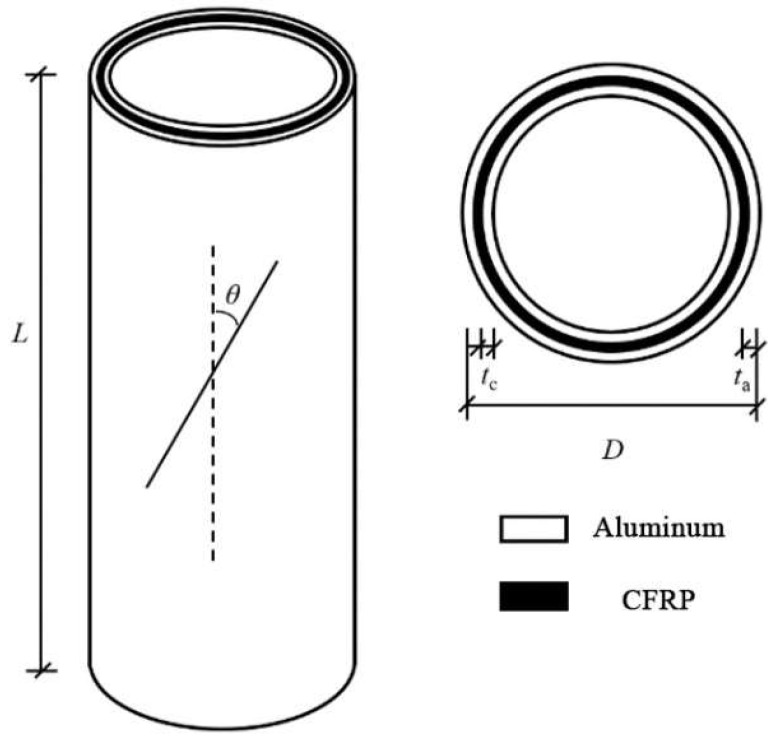
Composite Tube Dimension Annotations.

The specimen is shown in Figure 12. Axial compression tests were conducted on 27 CFRP-AL tubes and 3 aluminum alloy circular tubes. The cross-sectional dimensions and symbol annotations of the specimens are shown in Figure 13 and Table 10, where *n* represents the number of fiber winding layers and *λ* represents the slenderness ratio of the specimen. Each group of CFRP-AL tubes includes 3 duplicate specimens to verify the reliability of the test.

In the CFRP-AL tubes of this study, the carbon fibers are laid in an antisymmetric manner, and the angle between the fiber direction and the axial direction of the specimen is defined as the fiber winding angle *θ*. To ensure tight winding, the outermost layer of fibers in the specimens with a fiber winding angle of 0° is wound at an angle of 64°, while the winding angle of the other fiber layers is 0°.

#### 3.1.2. Test Device

**Figure 14:**
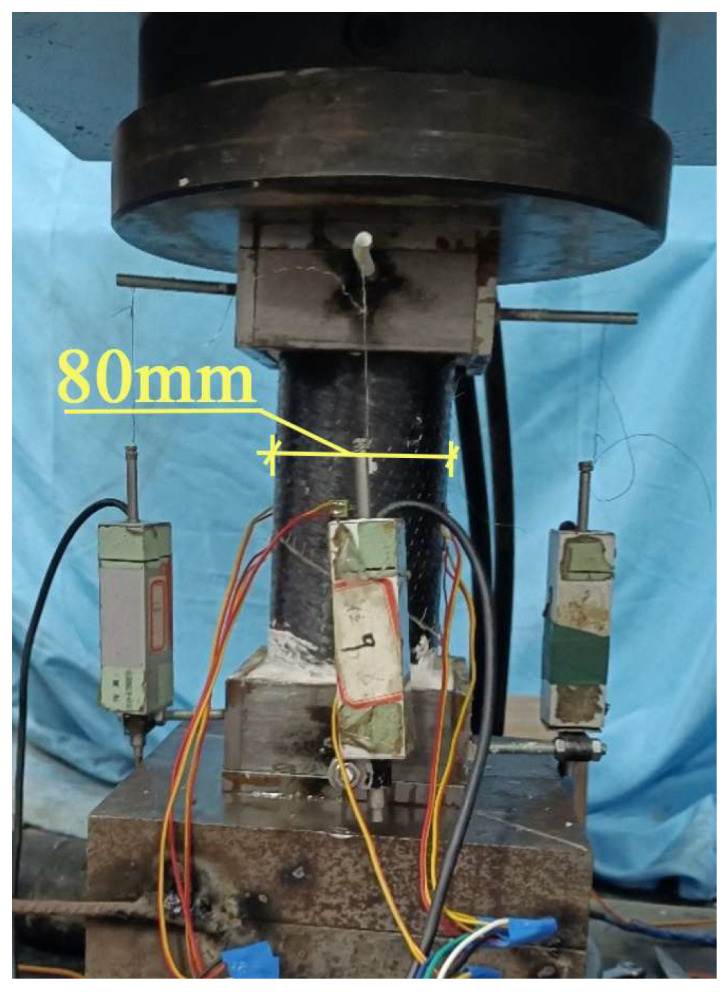
Axial Compression Test Loading Device.

The axial compression test of tubes is conducted on a universal testing machine. Specimen alignment primarily relies on geometric alignment, supplemented by strain alignment. A small preload (approximately 10% of the ultimate load) was applied in advance, and the position of the specimen was adjusted to ensure that the strain gauge readings at the mid-span were approximately consistent. The axial compression test uses displacement loading, with a loading speed set at 1 mm/min. The loading device is shown in Figure 14.

The ends of the specimens are anchored using α high-strength gypsum to achieve fixed connections at both ends. The prepared gypsum is poured into a detachable steel groove with a depth of 40 mm, and the specimen is inserted. Once the gypsum is solidified, the anchoring of the specimen ends is complete. After the test loading is finished, the steel groove is removed, and the gypsum is broken apart to observe the internal and external deformations of the CFRP-AL tubes from the specimen ends.

#### 3.1.3. Arrangement of Measurement Points

**Figure 15:**
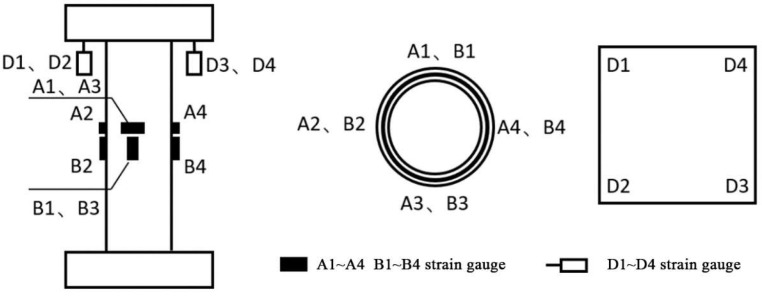
Axial Compression Test Device.

The arrangement of measurement points is shown in Figure 15. To measure the axial deformation of the specimen, four vertical displacement gauges are placed at the four corner points of the upper support. Additionally, eight strain gauges are arranged in a “T” shape on the mid span section, oriented perpendicular to each other, to measure the longitudinal and transverse strains at the mid-span cross-section.

### 3.2. Test Results and Analysis

#### 3.2.1. Failure Mode

**Figure 16:**
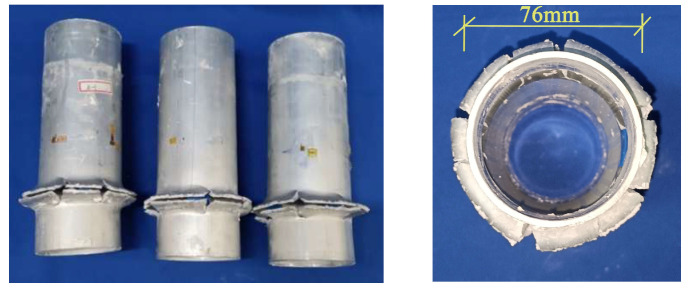
Failure Modes of Aluminum Alloy Specimens.

**Figure 17:**
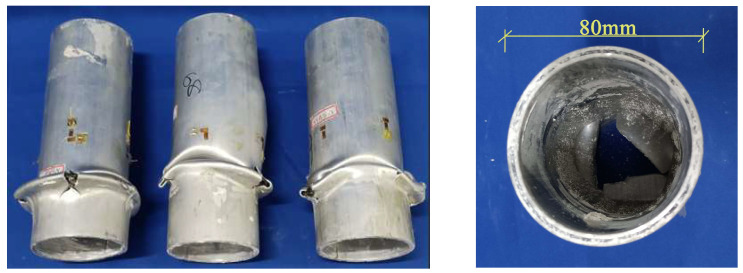
Failure Modes of N3A30 Specimen.

**Figure 18:**
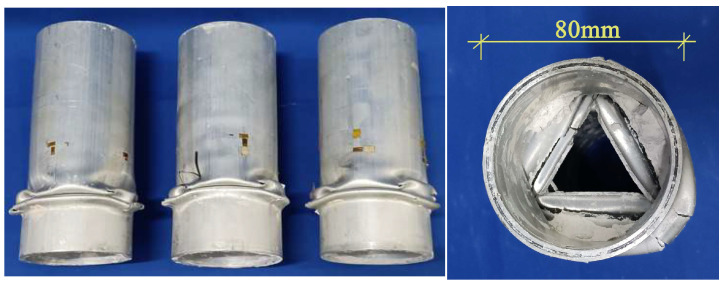
Failure Modes of N3A64 Specimen.

All short tube specimens experienced strength failure, with typical failure modes shown in Figure 16, Figure 17 and Figure 18. The specimen exhibited overlapping and cracking of the inner and outer aluminum tubes. The CFRP in the middle experienced crumple and fracture, and delamination occurred between the aluminum alloy and the CFRP. Most specimens began to fail from the ends, while a few started from the middle, which is related to the distribution of initial defects.

**Figure 19:**
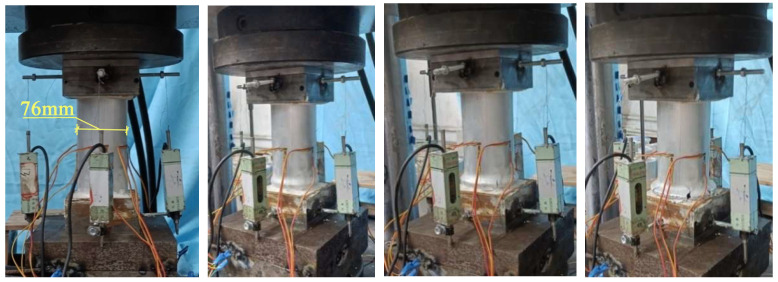
Loading Process of Aluminum Alloy Specimens.

Figure 19 illustrates the loading process of the aluminum alloy short tube. For the aluminum alloy short tube, as the axial compression load increases, buckling first appears at the ends. With continued loading, the buckling intensifies, leading to several longitudinal cracks, followed by fracturing. The upper and lower parts of the buckling crumple together, and cracks appear on the outermost circle of the buckling. With continued loading, the specimen will fracture at the circumferential crack, and the aluminum tube will crack and subsequently split longitudinally, at which point it will have almost no load-bearing capacity.

**Figure 20:**
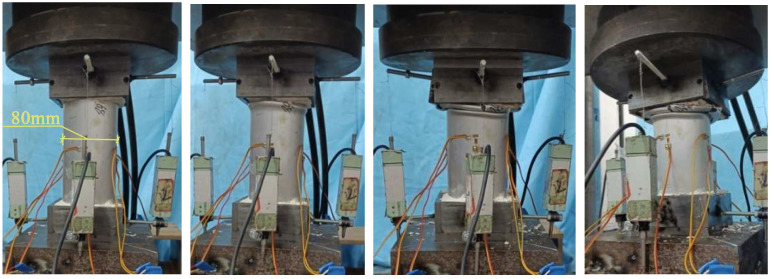
Loading Process of N3A30 Specimen.

Figure 20 shows the loading process of CFRP-AL tubes. For CFRP-AL tubes, the degree of damage varies depending on the fiber winding angle and the number of winding layers. Specimens with a fiber winding angle of 30° exhibit more longitudinal cracks than those with a 64° angle. Compared to the specimen with 4 layers of fiber winding, the specimen with 3 layers exhibited deeper overlapping deformation, a greater number of cracks, and larger crack sizes. Upon unloading and observation, it was found that in the localized damaged areas, the inner aluminum layer, carbon fiber layer, and outer aluminum layer were all separated from each other, with different crumpling directions and forms, leading to delamination between the layers.

For the N3A64 specimen, due to the enhanced effect of circumferential hooping, after buckling appears at the ends, only one or two longitudinal cracks appear or no cracks at all, and the cracks hardly propagate. The inner aluminum alloy tube at the failure location exhibited “diamond-shaped” overlapping, while the outer aluminum alloy tube displayed “accordion-shaped” overlapping. With continued loading, the inner aluminum tube will fracture at the folds of the “diamond-shaped” overlapping, while the outer aluminum tube does not develop circumferential cracks. Further loading will lead to new buckling, and the buckled areas will continue to crumple, allowing the specimen to maintain a higher level of load-bearing capacity.

Overall, in axial compression tests, after reaching the peak load, aluminum alloy tubes exhibit buckling, followed by longitudinal and circumferential cracks. Continued compression leads to longitudinal splitting, after which the tube almost loses its load-bearing capacity. In contrast, short CFRP-AL tubes, after reaching peak load in axial compression tests, show buckling with fewer or no longitudinal cracks and almost no circumferential cracks. Delamination occurs between layers, and continued compression results in progressive folding while still maintaining a relatively high load-bearing capacity. The failure mode of the specimen is influenced by the circumferential hooping effect of the CFRP.

#### 3.2.2. Ultimate Bearing Capacity

Table 11 presents the ultimate load capacity of short tube specimens in axial compression tests. In the table, *P_u,1_*, *P_u,2_*, and *P_u,3_* represent the maximum load capacity measured for three replicate specimens in the same group, while *P_u,avg_* is the average of the maximum load capacities measured for the specimens in the same group. *η*_s_ is the percentage increase in the average maximum load capacity of the specimens in the same group relative to the aluminum alloy specimens.

From the table, compared to pure aluminum alloy tubes, the ultimate load capacity of CFRP-AL tubes increased by 11% to 30%. Additionally, the improvement in ultimate load capacity of composite specimens is related to the fiber winding angle and the number of winding layers. Specimens with winding angles of 30° and 0° both show an increase in ultimate load capacity compared to specimens with 64° winding angles. The ultimate load capacity increases with more winding layers. This is because the ultimate load capacity of short tube specimens is primarily related to the equivalent axial strength of the specimens. As the fiber winding angle approaches the axial direction and the number of winding layers increases, the equivalent axial strength becomes greater, resulting in higher ultimate load capacity for the specimens.

#### 3.2.3. Test Curves

**Figure 21:**
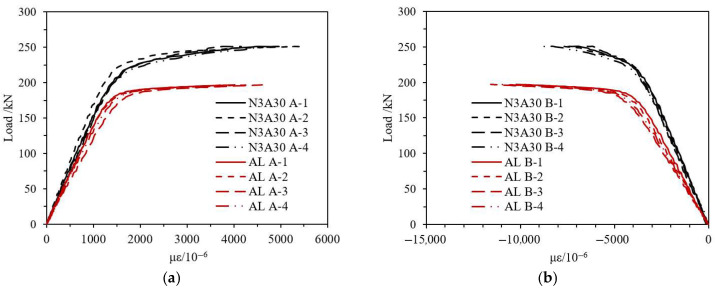
Load-Strain Curves of N3A30 and Aluminum Alloy Specimens. (**a**) Circumferential Strain Gauge A-1~A-4; (**b**) Axial Strain Gauge B-1~B-4.

**Figure 22:**
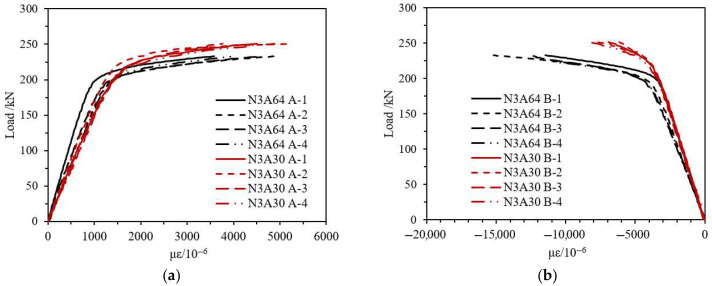
Load-Strain Curves of N3A64 and N3A30 Specimens. (**a**) Circumferential Strain Gauge A-1~A-4; (**b**) Axial Strain Gauge B-1~B-4.

Taking CFRP-AL tube N3A30 and aluminum alloy tube AL as examples, as shown in Figure 21, the load-strain curves of the specimens are compared. Under the axial compression load, the strain gauges arranged vertically at the mid-span recorded compressive strain, while those arranged laterally recorded tensile strain. This indicates that the specimens experienced axial shortening and radial expansion. Under the same axial load, both axial and radial strains of the composite specimens are smaller than those of the aluminum alloy specimens, indicating that CFRP can effectively constrain the deformation of the specimens. From Figure 22, the fiber winding angle affects the axial and radial strains of the specimens. Specimens with a winding angle of 30° have greater equivalent axial strength, resulting in smaller axial strain under the same axial load compared to specimens with a winding angle of 64°.

**Figure 23:**
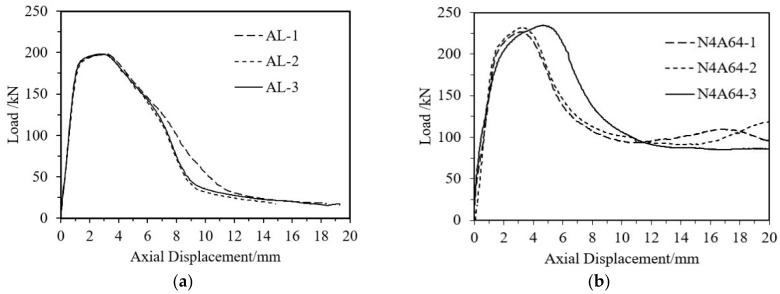
Repeatability Test Results for Selected Specimens. (**a**) AL; (**b**) N4A64.

Figure 23, using the CFRP-AL tube N4A64 and aluminum alloy tube as examples, demonstrates that the results of the repeatability tests have good consistency. Combined with the previously mentioned failure modes, this further demonstrates the reliability of the specimen quality and the testing system used in this study.

**Figure 24:**
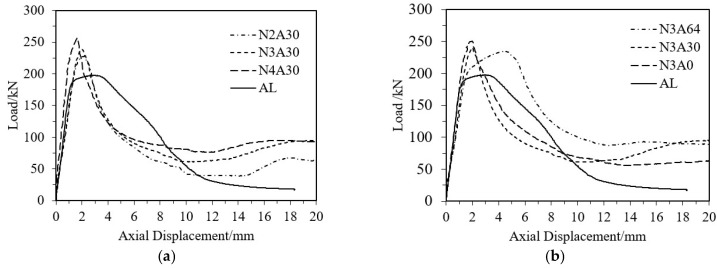
Comparison of Load–Axial Displacement Curves for Selected Specimens. (**a**) Comparison of Different Fiber Layer Thicknesses; (**b**) Comparison of Different Winding Angles.

**Figure 25:**
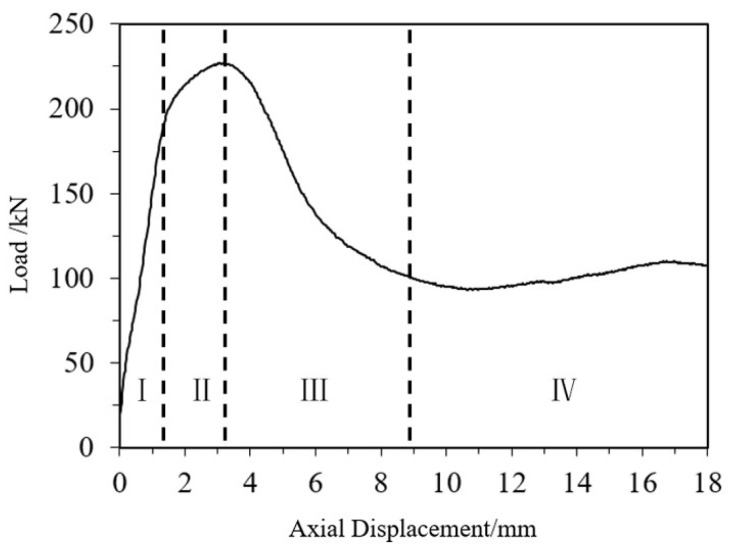
Stages of Axial Compression Testing for Composite Specimens.

The load–axial displacement curves of CFRP-AL tubes and aluminum alloy tubes were compared, as shown in Figure 24. By analyzing the failure modes of the specimens and the load-strain curves, it can be concluded that the axial compression process of CFRP-AL tubes can be divided into four stages, as illustrated in Figure 25. Stage I: Elastic Stage—At the initial loading stage, the load–displacement curve is almost linear, and the specimen mainly undergoes elastic deformation. The load is primarily borne by the aluminum alloy, which has greater stiffness. CFRP deforms together due to bonding but only bears a small portion of the load due to its lower stiffness. Stage II: Plastic Deformation Stage—This stage lasts from the end of the elastic phase until the specimen reaches the peak load. The aluminum alloy enters the plastic phase. The tube undergoes lateral expansion. The circumferential confinement effect of the CFRP can limit the occurrence and development of aluminum tube buckling. As a result, specimens with a fiber winding angle of 64° have a longer plastic deformation stage, while those with a winding angle of 30° have a shorter plastic deformation stage. Nevertheless, specimens with winding angles of 30° and 0° have greater equivalent axial strength, leading to a higher ultimate load capacity for the composite components. Stage III: Buckling Stage—After the specimen reaches its ultimate load capacity, noticeable buckling occurs at the ends, and the load decreases rapidly. Stage IV: Progressive folding Stage—Due to the circumferential confinement effect of the CFRP, the longitudinal splitting of the aluminum alloy is restricted. Therefore, after the first layer of crumple is completed, the CFRP-AL tubes can continue to undergo a second layer and subsequent crumple. Moreover, due to the circumferential confinement effect of the CFRP, the specimen can maintain a relatively high level of load-bearing capacity during this process.

For aluminum alloy tubes, the axial compression process is similar and can also be divided into the same four stages. The difference is that the ultimate load capacity of aluminum alloy components is lower. After subsequent buckling, longitudinal cracks will develop, leading to “blossoming” longitudinal splitting, which results in almost complete loss of load-bearing capacity.

In summary, it can be observed that in the axial compression tests, CFRP primarily serves two major functions:

(1) Through the equivalent axial strength of CFRP, it directly increases the ultimate load capacity of the composite specimens;
(2) Through the circumferential confinement effect of CFRP, it limits the initiation and development of crushing deformation after reaching the peak load, thereby enhancing the subsequent load-bearing capacity.

The fiber winding angle and the number of fiber layers determine the proportion and magnitude of these two functions.

## 4. Finite Element Analysis

### 4.1. Finite Element Model

**Figure 26:**
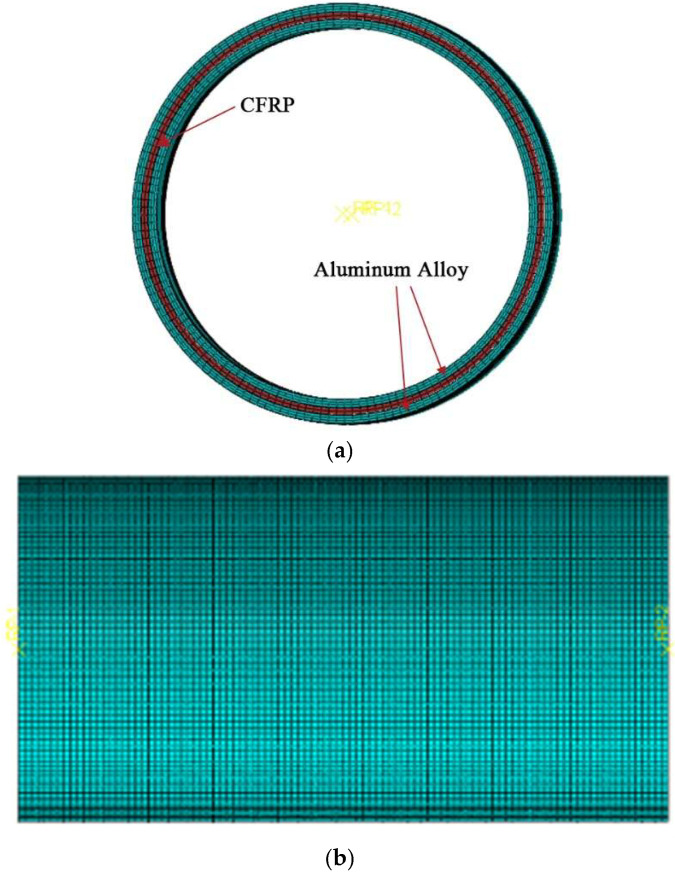
Finite Element Model for the Axial Compression of the CFRP-AL Tube. (**a**) Specimen Cross-Section; (**b**) Short Tube Specimen.

The finite element model is shown in Figure 26. In this study, the ABAQUS/Explicit module was chosen for the axial compression analysis of CFRP-AL tubes. The CFRP constitutive model utilizes the VUMAT user subroutine suitable for explicit analysis. The loading time is set to 0.05 s, with a smooth amplitude curve (Smooth step) applied to the load. Additionally, a uniform mass scaling of 100 was applied to all elements to balance computational efficiency and accuracy. At this point, kinetic energy is less than 5% of the total internal energy, and the influence of inertial forces on the analysis results can be neglected. In addition, the initial imperfection of the specimen adopts the lowest order buckling mode. The initial imperfection is determined through back-calculation using axial strain gauges around the specimen and applied to the model.

The finite element model for axial compression of CFRP-AL tubes consists of three parts: the inner aluminum alloy tube, the middle CFRP tube, and the outer aluminum alloy tube.

The inner and outer aluminum alloys are simulated using an isotropic elasto-plasticity model, employing the Johnson–Cook hardening criterion and its damage evolution criteria. The material parameters can be found in Section 2.1. The interfaces between CFRP layers and between CFRP and aluminum alloys are modeled using surface-based cohesive behavior, with the cohesive contact parameters detailed in Section 2.3.

CFRP’s failure modes under static loads are relatively complex. Existing research indicates that the Puck failure criterion is quite accurate in predicting matrix compression failure modes, while the Hashin failure criterion is more straightforward for predicting the other three types of failure modes. ABAQUS only includes a 2D Hashin failure criterion and has not embedded a 3D Hashin failure criterion, which cannot be used for 3D solid elements. This study selects the appropriate failure criteria mentioned earlier. A user subroutine for the material constitutive model (VUMAT) based on the ABAQUS/Explicit module was developed.

**Figure 27:**
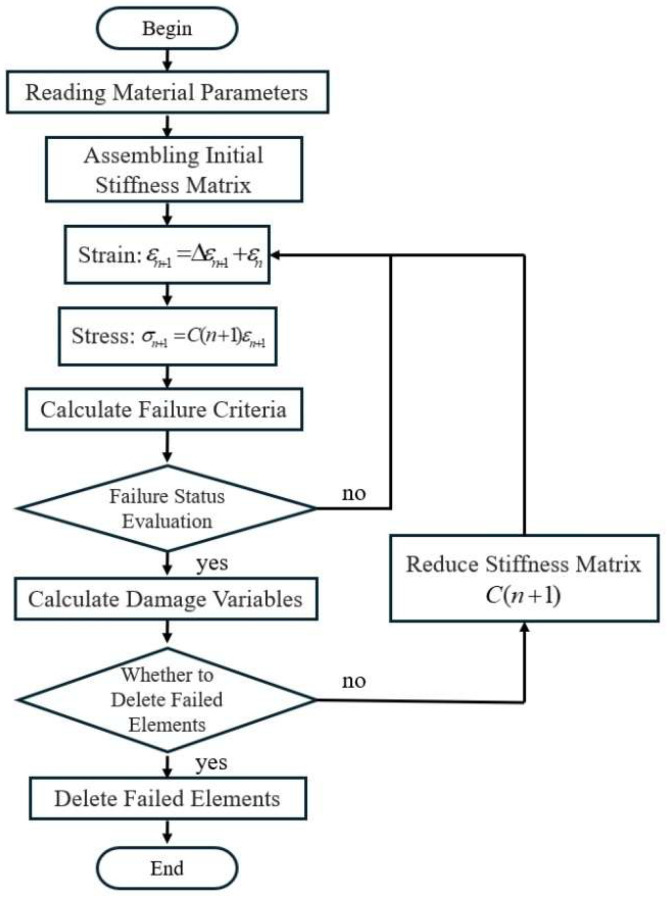
User Subroutine Calculation Flowchart.

The specific algorithm flow is as Figure 27:

Damage variables are used as the criterion for deleting failed elements, as shown in Formulas (13)–(16). The calculation of damage variables considers the compressive damage of fibers, compressive damage of the matrix, tensile damage of fibers, and tensile damage of the matrix. The main criterion for these damage variables is the strain of the material. When a damage variable reaches the threshold value, the element is deleted. At this point, the plastic strains of both the fibers and the matrix are already sufficiently large, and the stiffness is sufficiently small. When there is a certain degree of damage but the threshold for element deletion is not reached, the stiffness is reduced according to Formula (25). After an element is deleted, its stiffness is reduced to 0.

CFRP material parameters are detailed in Section 2.2.

To improve computational efficiency, both ends are fixed instead of using support forms. The load is applied by imposing axial displacement at one end of the component. The inner aluminum alloy tube, the middle CFRP tube, and the outer aluminum alloy tube all use C3D8R elements, with hourglass control and element deletion enabled.

### 4.2. Finite Element Calculation Results

Taking aluminum alloy tubes and CFRP-AL tubes with different winding angles and layers as examples, the failure modes and load–axial displacement curves from finite element simulations were compared with experimental results to verify the rationality of the finite element model.

**Figure 28:**
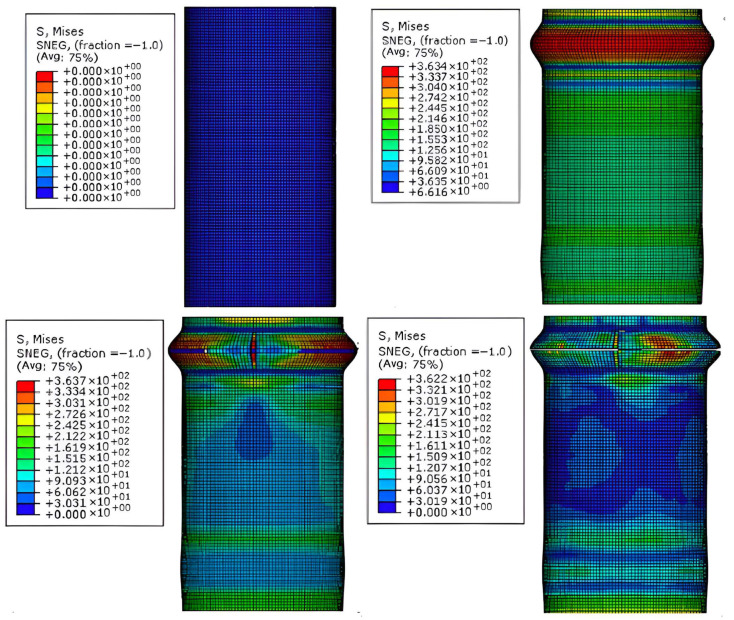
Finite Element Loading Process of Aluminum Alloy tube.

**Figure 29:**
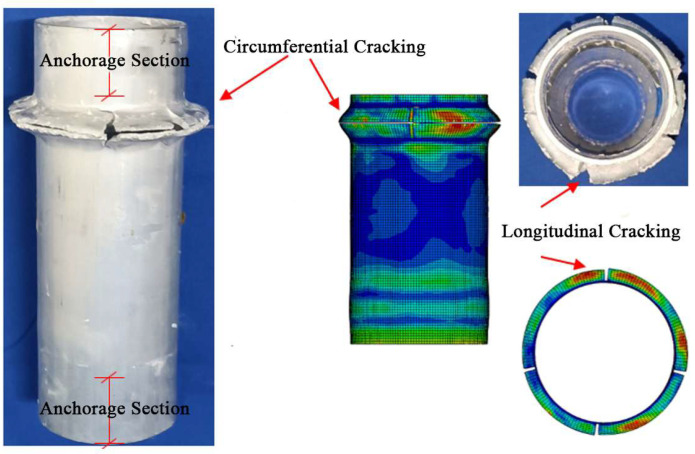
Comparison of Finite Element and Experimental Failure Modes of Aluminum Alloy tube.

As shown in Figure 28, the failure process of the aluminum alloy specimen’s finite element model closely matches the experimental failure state depicted in Figure 16. With increasing load, “elephant foot” buckling appears at the ends, followed by several longitudinal cracks. The upper and lower parts of the buckled section collapse together, and circumferential cracks appear on the outermost circle. Figure 29 compares the finite element and experimental failure modes of the aluminum alloy specimen, where both show longitudinal and circumferential cracking at the buckling locations.

**Figure 30:**
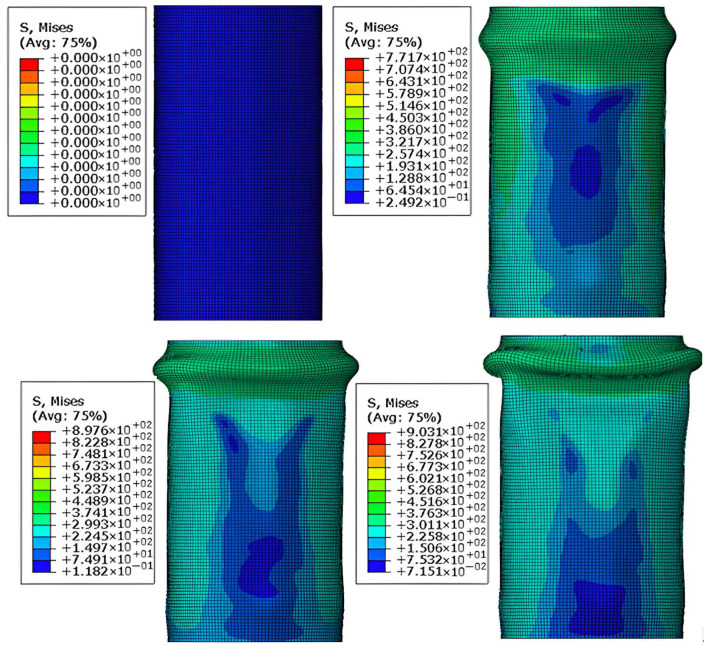
Finite Element Loading Process of CFRP-AL tube N3A64.

**Figure 31:**
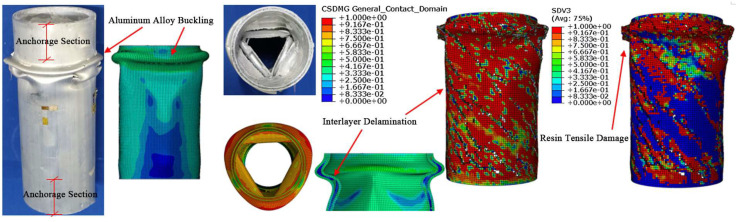
Comparison of Finite Element and Experimental Failure Modes of CFRP-AL tube N3A64.

As shown in Figure 30 and Figure 31, the failure process and final failure mode of the CFRP-AL tube N3A64’s finite element model are basically consistent with the experimental results. After “elephant foot” buckling appears at the ends, no cracks occur. The inner aluminum alloy tube exhibits “diamond-shaped” buckling, the outer aluminum alloy tube shows “accordion-style” buckling, and delamination occurs between the inner aluminum alloy layer, the carbon fiber layer, and the outer aluminum alloy layer.

**Figure 32:**
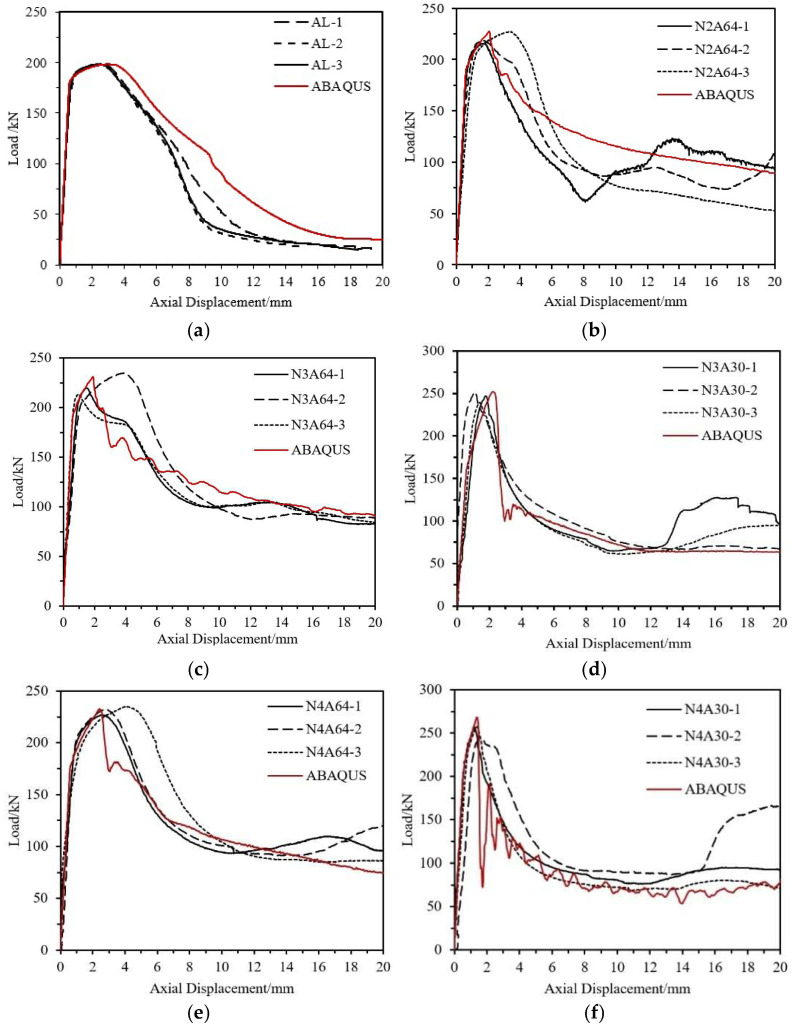
Comparison of Load–Axial Displacement Curves between Experimental and Finite Element Results for Short Tube Specimens. (**a**) AL Specimen; (**b**) N2A64 Specimen; (**c**) N3A64 Specimen; (**d**) N3A30 Specimen; (**e**) N4A64 Specimen; (**f**) N4A30 Specimen.

In addition to failure modes, the load–axial displacement curve is also an important indicator for evaluating the accuracy of the specimen’s finite element model. A comparison between the finite element results and experimental results is shown in Figure 32. It can be seen that for specimens with different fiber winding angles and numbers of layers, the finite element model can effectively simulate the axial compression test results, including initial stiffness, peak load, descending trend, and stable load.

### 4.3. Mechanism Analysis of CFRP-AL Tubes Under Axial Force

**Figure 33:**
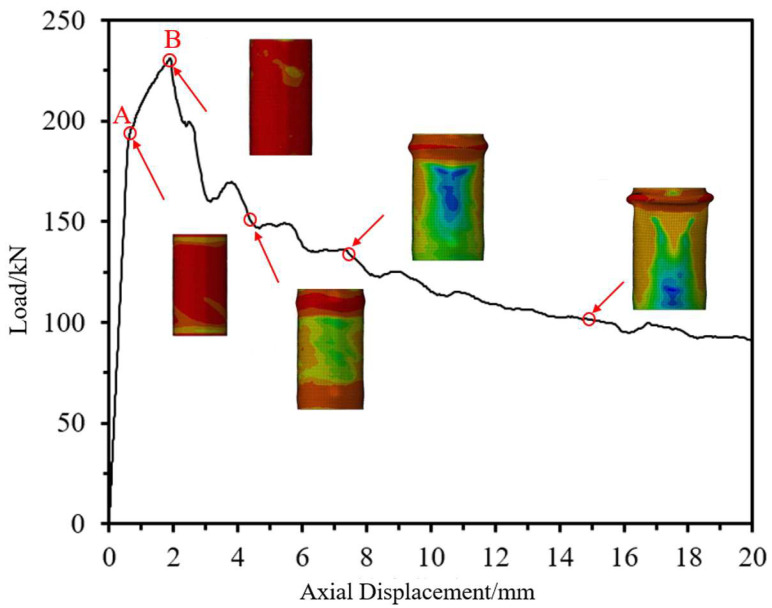
Failure Process of Finite Element Model for Composite Specimen N3A64.

To further analyze the axial force mechanism of CFRP-AL tubes, this section uses the finite element model, taking N3A64 as an example, to analyze the stress state and damage state throughout the failure process of the finite element model. The focus is on examining the stress state, contact state, and damage state of the interfaces between aluminum alloy, CFRP, and between the aluminum alloy and CFRP layers. As discussed earlier, the axial compression process of CFRP-AL tubes can be divided into four stages: elastic stage, plastic deformation stage, buckling stage, and progressive folding stage. The stress contour maps corresponding to each stage of the specimen are shown in Figure 33.

**Figure 34:**
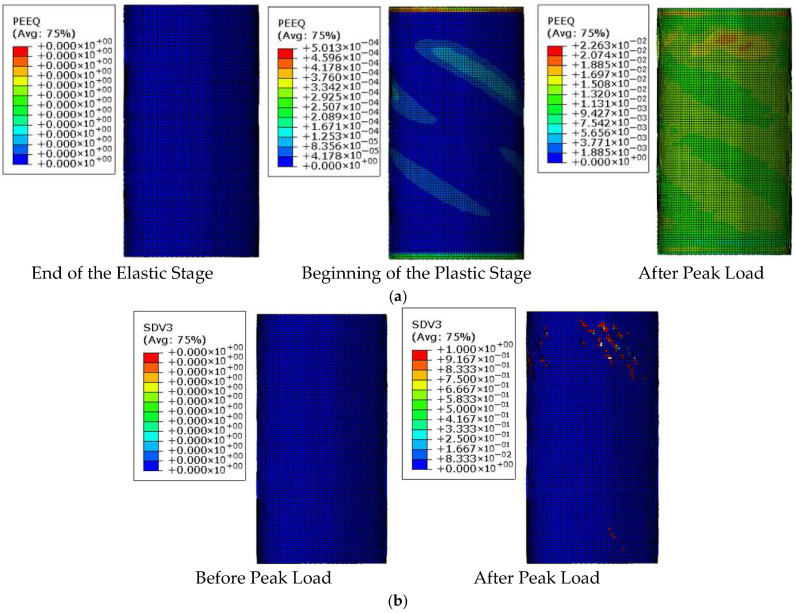
Indicators of Failure Stages in the Finite Element Model for Specimen N3A64. (**a**) Marked by Aluminum Alloy Entering Plasticity; (**b**) Marked by CFRP Resin Damage.

In practical engineering, more attention is given to the state of the specimen just as it begins to enter the plastic stage (Point A in Figure 33) and at the peak load (Point B in Figure 33). Therefore, the stress and damage states at these two typical moments are analyzed, as shown in Figure 34.

**Figure 35:**
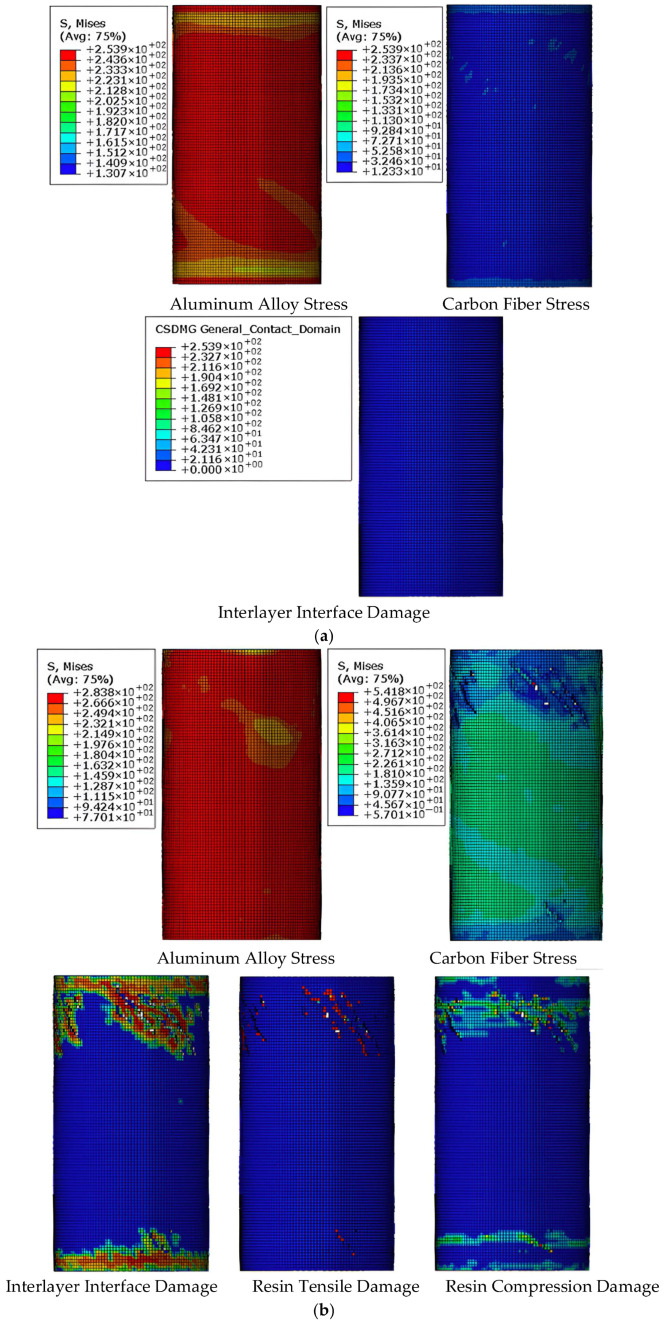
Special Moments in the Finite Element Model for Specimen N3A64. (**a**) Moment at the End of the Elastic Stage (Moment A); (**b**) Moment at Peak Load (Moment B).

Point A is marked by the aluminum alloy material entering the plastic stage. During the elastic stage, the aluminum alloy has not yet entered the plastic stage. It begins to enter plasticity as the specimen transitions into the plastic stage, as shown in Figure 34a), where the axial stiffness of the specimen starts to decrease. In the elastic stage, both the aluminum alloy and CFRP are under longitudinal compression along their entire length, with the aluminum alloy experiencing significant longitudinal compressive stress, while the CFRP stress level is not high, and the circumferential stress is relatively small. No delamination damage is observed at the interface between layers, as shown in Figure 35a). During this stage, the load–displacement curve is linear until the end of the elastic stage.

Point B is marked by damage occurring in the resin within the CFRP. In the plastic stage, the aluminum alloy enters plasticity, leading to increased axial displacement for the same load increment. The stress in the CFRP begins to increase rapidly, but no damage has been observed yet. Upon reaching the peak load, damage occurs at the interface between layers, and the resin experiences significant tensile stress, leading to damage, as shown in Figure 34b). The load begins to decrease rapidly, and axial displacement develops quickly. During this stage, no obvious buckling is observed in the aluminum alloy. However, during the buckling stage, the aluminum alloy experiences “elephant foot” buckling due to high compressive stress.

In summary, during the elastic and plastic stages, the CFRP in CFRP-AL tubes primarily enhances the ultimate load-bearing capacity through its equivalent axial reinforcement effect. In the buckling stage, the CFRP mainly acts through its hoop confinement effect to restrict the initiation and development of progressive folding deformation after the peak load, thereby improving the subsequent load-bearing capacity.

## 5. Axial Compression Load-Bearing Capacity Formula

### 5.1. Axial Compression Parameter Analysis of CFRP-AL Tubes

Before deriving the calculation formula for the ultimate bearing capacity, it is necessary to determine the influence of each parameter on the ultimate bearing capacity through parameter analysis.

#### 5.1.1. Influence of Initial Imperfections

**Figure 36:**
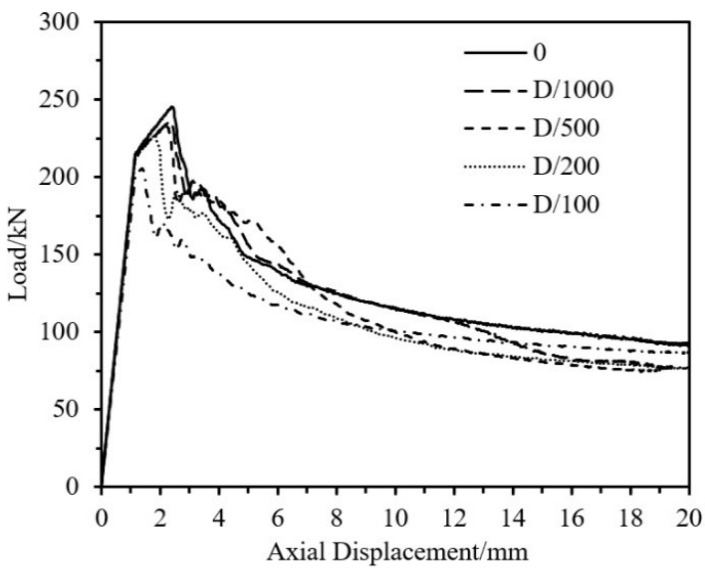
Load–axial Displacement Curves of Specimens under Different Imperfection.

Applying different magnitudes of initial imperfections to the same specimen: no imperfection, D/1000, D/500, D/200, and D/100, with specimen parameters as shown in Table 12; the resulting load–axial displacement curves are shown in Figure 36. It can be seen from the figure that the larger the initial imperfection, the lower the ultimate load-bearing capacity of the specimen, and the smaller the axial displacement corresponding to the ultimate load. This is because the evolution of the initial imperfection leads to the premature failure of the specimen. According to GB/T4436-2012 “Wrought aluminum and aluminum alloy tubes—Dimensions and deviations“ [45], when the outer diameter of aluminum alloy tubes is 80~100 mm, the permissible deviation for high precision tubes is ±0.38 mm. Therefore, for ease of comparison, the magnitude of initial imperfection for the parameter analysis model in this section is uniformly set to D/200.

#### 5.1.2. Influence of Component Dimensions

**Figure 37:**
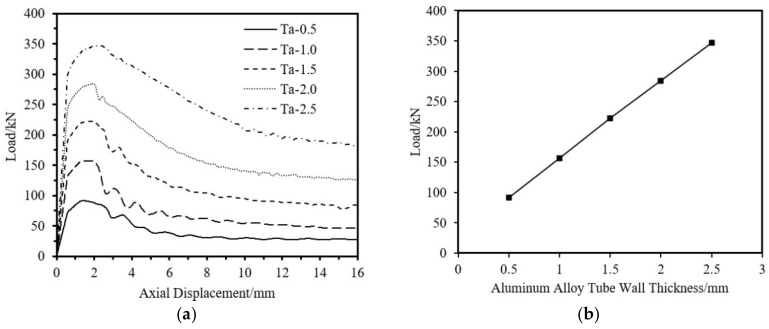
Impact of Aluminum Alloy Thickness on the Axial Compression Load-Bearing Performance of Specimens. (**a**) Load–Axial Displacement Curve; (**b**) Ultimate Load.

**Figure 38:**
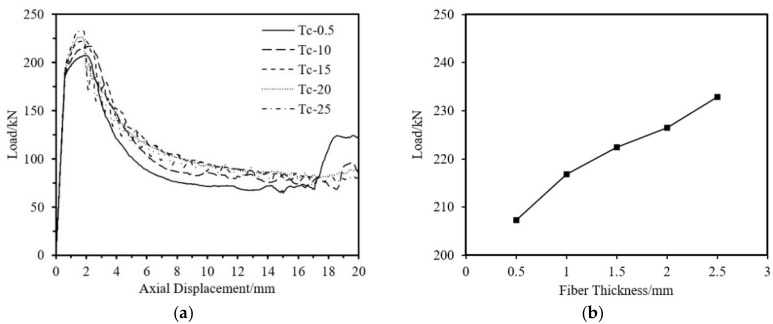
Impact of Fiber Thickness on the Axial Compression Load-Bearing Performance of Specimens. (**a**) Load–Axial Displacement Curve; (**b**) Ultimate Load.

**Figure 39:**
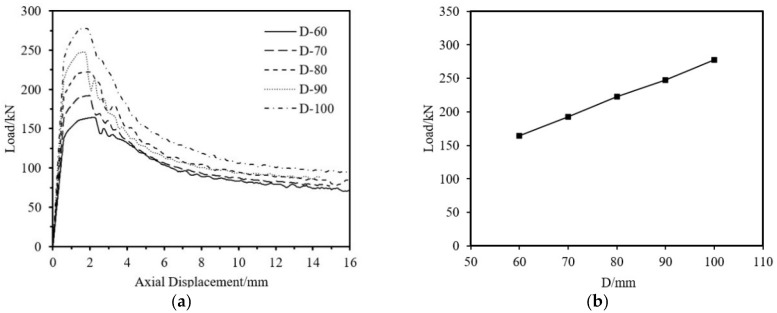
Impact of Outer Diameter on the Axial Compression Load-Bearing Performance of Specimens. (**a**) Load–Axial Displacement Curve; (**b**) Ultimate Load.

**Figure 40:**
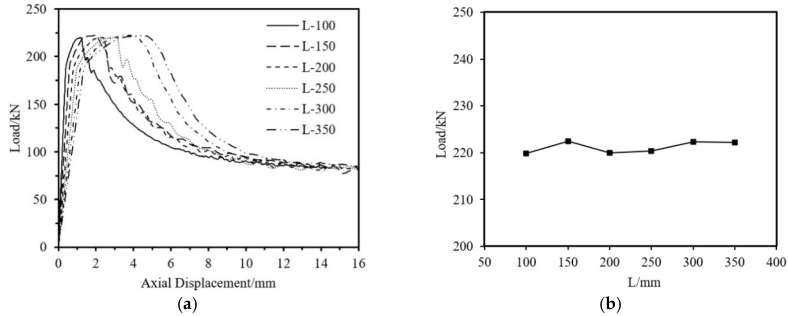
Impact of tube Length on the Axial Compression Load-Bearing Performance of Specimens. (**a**) Load–Axial Displacement Curve; (**b**) Ultimate Load.

The impact on ultimate load-bearing capacity from four aspects—aluminum tube thickness, fiber winding thickness, outer diameter, and tube length is analyzed. The specimen parameters are shown in Table 13. As shown in Figure 37, Figure 38 and Figure 39, the ultimate load-bearing capacity of the specimen is approximately proportional to the tube outer diameter, aluminum tube thickness, and fiber winding thickness. As shown in Figure 40, the ultimate load-bearing capacity changes little with the tube length.

#### 5.1.3. Influence of Fiber Angle

**Figure 41:**
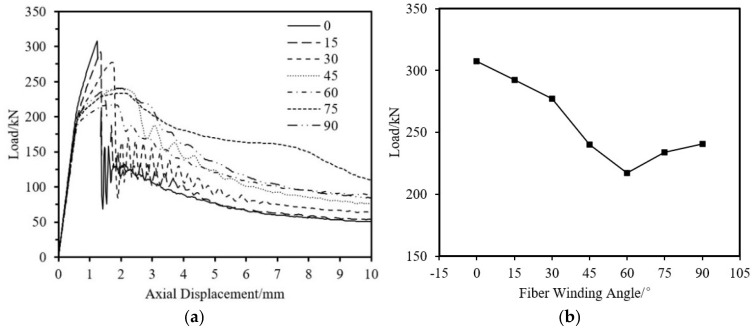
Impact of Fiber Angle on the Axial Compression Load-Bearing Performance of Specimens. (**a**) Load–Axial Displacement Curve; (**b**) Ultimate Load.

The specimen parameters are shown in Table 14. The ultimate load-bearing capacity of short tube specimens is significantly affected by the fiber winding angle. As shown in Figure 41, within the fiber angle range of 0° to 45°, the ultimate load of the specimen decreases significantly as the fiber winding angle increases. From 45° to 90°, the ultimate load of the specimen increases slightly with the increase in fiber winding angle, similar to the pattern observed in experiments. The reason is that the ultimate load-bearing capacity of the CFRP-AL tubes is largely influenced by the equivalent axial reinforcement effect and partially by the circumferential hoop effect of the carbon fibers. As the fiber winding angle approaches the axial direction, the equivalent axial effect becomes stronger; as it approaches the circumferential direction, the hoop effect becomes stronger. Under the combined influence of the two, the curve of ultimate bearing capacity versus fiber winding angle shows a trend of first decreasing and then increasing.

#### 5.1.4. Influence of Fiber Volume Ratio

**Figure 42:**
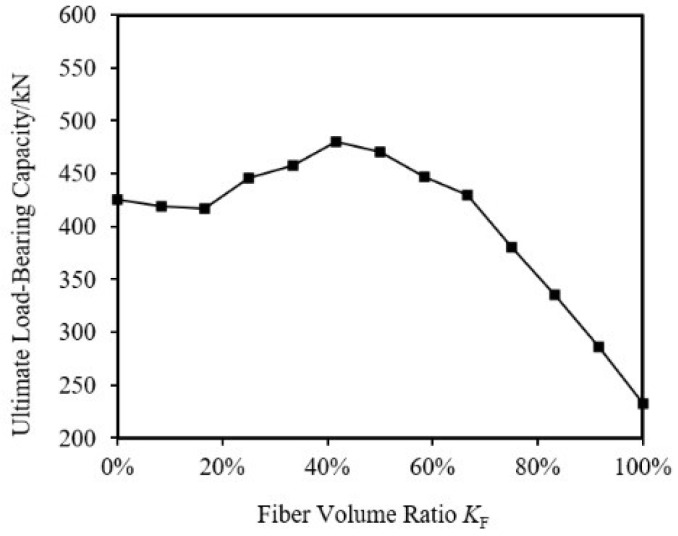
Impact of *K*_F_ on the axial compression ultimate load-bearing capacity of specimens.

**Figure 43:**
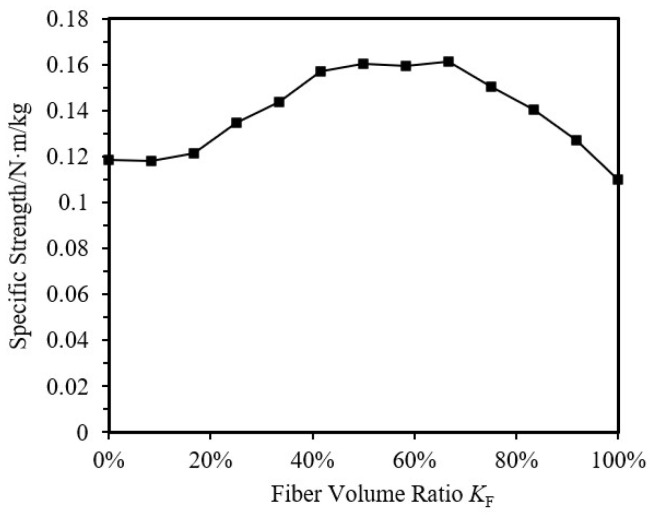
Impact of *K*_F_ on the axial compression specific strength of specimens.

By maintaining the same outer diameter and thickness of the specimens and changing the fiber volume ratio *K*_F_ (i.e., the ratio of CFRP volume to the total volume of the specimen), a series of specimens transitioning from pure aluminum alloy to pure CFRP were analyzed for their ultimate load-bearing capacity. The specimen parameters are shown in Table 15, and the results for ultimate load-bearing capacity and specific strength (the ratio of compressive strength to equivalent density) are shown in Figure 42 and Figure 43.

From the figures, it can be observed that the specimen mass decreases almost linearly as K_F_ increases. The ultimate load-bearing capacity of the specimen first increases and then decreases with K_F_, reaching a maximum at 41.67%. In the range of 25.00% to 66.67% fiber volume ratio, the ultimate load-bearing capacity of the CFRP-AL tubes is greater than that of pure aluminum alloy tubes. The specific strength of the specimen first increases and then decreases with *K*_F_, with CFRP-AL tubes in the fiber volume ratio range of 41.67% to 66.67% exhibiting higher specific strength. The specific strength of CFRP-AL tubes is greater than that of both pure aluminum alloy and pure CFRP components.

### 5.2. Load-Bearing Capacity Formula

To facilitate application in the engineering field, this section proposes a formula for calculating the ultimate load-bearing capacity of short tube specimens based on previous analyses. According to the analysis of the axial force mechanism of CFRP-AL tubes in Section 4.3, at peak load, the aluminum alloy is fully plastic across the entire section, and the CFRP has just reached its ultimate strength, resulting in damage. When CFRP-AL tube reaches its ultimate load, no delamination occurs within the CFRP layer or at the interface between the aluminum alloy and CFRP, thus the effect of interlayer delamination failure within the CFRP layer is neglected.

Therefore, this section introduces an equivalent cross-sectional area, equating CFRP to aluminum alloy material, and considers the yield load of the equivalent cross-section as the ultimate load-bearing capacity of the tubes. The following simplified calculation formula is proposed:

(31) $$P_{u}=A_{\mathrm{eq}}f_{0.2}$$

Equivalent Cross-Sectional Area $A_{\mathrm{eq}}$ is:

(32) $$A_{\mathrm{eq}}=\frac{\sum_{i=1}^{n}E_{Ci}A_{Ci}+\sum_{i=1}^{n}E_{A}A_{Ai}}{E_{A}}$$

The above formula can be written as:

(33) $$P_{u}=(\sum_{i=1}^{n}E_{Ci}A_{Ci}+\sum_{i=1}^{n}E_{A}A_{Ai})\frac{f_{0.2}}{E_{A}}$$

$E_{Ci}$—Axial Elastic Modulus of the i-th Layer of CFRP;
$A_{Ci}$—Cross-Sectional Area of the i-th Layer of CFRP;
$E_{A}$—Elastic Modulus of Aluminum Alloy;
$A_{Ai}$—Cross-Sectional Area of the i-th Layer of Aluminum Alloy;
$f_{0.2}$—Yield Strength of Aluminum Alloy.

As shown in Table 16, the calculated results are compared with the experimental results and finite element simulation results. Except for the N3A0 and N4A0 specimens, where the error between the experimental results and the calculated values is within 20%, the errors for the other specimens are all within 10%. This discrepancy may be due to the immaturity of the manufacturing process, leading to slightly larger initial imperfection when laying the 0° fiber layer. The error between the finite element results and the calculated values is relatively small, within 5%. Therefore, the simplified calculation formula can effectively estimate the ultimate load-bearing capacity of CFRP-AL tubes. Feng et al. [26] conducted tests by wrapping CFRP around the outside of aluminum alloy tubes, and finally obtained conclusions similar to our ultimate bearing capacity calculation formula, which further proves the reliability of our conclusions.

## 6. Conclusions

To investigate the failure modes and response patterns of CFRP-AL tubes under axial compression, this study conducted axial compression tests and finite element analysis on 27 CFRP-AL tubes with relatively small slenderness ratios and 3 aluminum alloy circular tubes. The main content and conclusions are as follows:

1. The typical failure mode of the specimen is strength failure. Compared with the pure aluminum alloy tube, the ultimate bearing capacity of the CFRP-AL tubes is increased by 11% to 31%.
2. The axial compression process of short CFRP-AL tubes can be divided into four stages: elastic stage, plastic deformation stage, buckling stage, and progressive folding load-bearing stage. The role of CFRP in this process is two-fold: firstly, it directly enhances the ultimate load-bearing capacity of the CFRP-AL tubes through equivalent axial strength; secondly, it restricts the initiation and development of folding deformation after peak load through circumferential hoop action. The proportion and magnitude of these two effects are determined by the fiber winding angle and the number of fiber layers.
3. Finite element simulations of axial compression specimens were conducted using user material subroutine, considering the effects of initial imperfection, fiber winding angle, fiber thickness, aluminum alloy tube wall thickness, outer diameter, and CFRP volume ratio. The failure patterns of CFRP-AL tubes under axial compression were obtained. The ultimate load-bearing capacity of CFRP-AL tubes first increases and then decreases with the fiber volume ratio *K*_F_, reaching its maximum at 41.67%. The ultimate load-bearing capacity of CFRP-AL tubes within the fiber volume ratio range of 25.00% to 66.67% is greater than that of pure aluminum alloy tubes. The specific strength of the tubes first increases and then decreases with *K*_F_, and the specific strength of CFRP-AL tubes within the range of 41.67% to 66.67% is relatively high. The specific strength of CFRP-AL tubes is greater than that of pure aluminum alloy tubes and pure CFRP tubes.
4. Based on the concept of equivalence and strength theory, a formula for calculating the ultimate load-bearing capacity of short tube strength failure was established. The calculated results are in good agreement with experimental and numerical results. The reliability of the formula has also been confirmed by comparison with other literature.

## Figures and Tables

**Table 1:** 6061-T6 Aluminum alloy (3.0 mm thickness) mechanical properties.

Specimen Number	Thicknesses *t*/mm	Yield Strength *σ*_0.2_/MPa	Ultimate Tensile Strength *σ*_u_/MPa	Modulus of Elasticity *E*/MPa	Yield Strain *ε*_y_/με	Fracture Strain *ε*_u_/με
AL-A1	3.0	266	320	64,303	6125	96,658
AL-A2	3.0	265	314	65,714	6094	83,313
AL-A3	3.0	266	317	66,354	6135	99,955
AL-A4	3.0	266	319	63,553	6142	103,852
avg	-	266	318	64,981	6124	95,945

**Table 2:** 6061-T6 Aluminum alloy (1.5 mm thickness) mechanical properties.

Specimen Number	Thicknesses *t*/mm	Yield Strength *σ*_0.2_/MPa	Ultimate Tensile Strength *σ*_u_/MPa	Modulus of Elasticity *E*/MPa	Yield Strain *ε*_y_/με	Fracture Strain *ε*_u_/με
AL-B1	1.5	259	295	63,013	6211	66,523
AL-B2	1.5	260	299	63,490	6124	79,121
AL-B3	1.5	258	297	62,792	6131	87,595
AL-B4	1.5	258	291	62,996	6109	81,042
avg	-	259	296	63,073	6144	78,570

**Table 3:** J–C Hardening Model Parameters of 6061-T6 Aluminum Alloy.

*A*/MPa	*B*/MPa	*n*	*C*	*m*
246	151	0.39	0.1	1.34

**Table 4:** Fracture Model Parameters of 6061-T6 Aluminum Alloy.

*D*_1_	*D*_2_	*D*_3_	*D*_4_	*D*_5_
−0.877	1.1	−0.47	0.01	1.6

**Table 5:** Properties of Carbon Fiber Fabric.

Thickness *t*/mm	Tensile Strength *f*_t/_MPa	Elastic Modulus *E*_f/_GPa	Elongation *δ*_t/_%
0.167	3543	249	1.4

**Table 6:** Properties of Epoxy Resin.

Tensile Strength *f*_t_/MPa	Compressive Strength *f*_c_/MPa	Elastic Modulus *E*/GPa	Elongation/%
54.4	100.7	3.3	2.1

**Table 7:** CFRP Engineering Constants.

*E*_1_ /MPa	*E*_2_ /MPa	*E*_3_ /MPa	*v*_12_	*v*_13_	*v*_23_	*G*_12_ /GPa	*G*_13_ /GPa	*G*_23_ /GPa
85,593	7982	7982	0.37	0.37	0.37	2365	2365	2476

**Table 8:** CFRP Directional Strengths and Fracture Energy.

*X*_t_ /MPa	*X*_c_ /MPa	*Y*_t_ = *Z*_t_/MPa	*Y*_c_ = *Z*_c_/MPa	*S*_12_ = *S*_13_ /MPa	*S*_23_ /MPa	$G_{1C}^{t}$ N/mm	$G_{1C}^{c}$ N/mm	$G_{2C}^{t}=G_{3C}^{t}$ N/mm	$G_{2C}^{c}=G_{3C}^{c}$ N/mm
1220	870	36.2	67	55	55	76	34	0.6	2.1

The subscripts *t* and *c* represent tensile state and compressive states, *X*, *Y*, and *Z* represent the strengths in different directions, *S* is shear strength, and *G* is fracture energy.

**Table 9:** Interlayer Interface Constitutive Parameters.

*K_nn_* GPa/mm	*K_ss_* GPa/mm	*K_tt_* GPa/mm	*T_n_* /MPa	*T_s_* /MPa	*T_t_* /MPa	*G_n_* N/mm	*G_s_* N/mm	*G_t_* N/mm	*η*
100	100	100	60	80	80	0.352	1.45	1.45	2

Where *K* is the interface stiffness, *T* is the interface strength, and *G* is the interface fractured energy; *n*, *s*, and *t* represent the normal and shear directions.

**Table 10:** Parameters of Specimens for the Axial Compression Test of Short Tubes.

Specimen Number	*L*/mm	*D*/mm	*t*_a_/mm	*t*_c_/mm	*θ*/°	*n*	λ	Specimen Type
N2A64	150	80	1.5	1.0	64	2	5.57	Composite Tube
N2A30	150	80	1.5	1.0	30	2	5.57	Composite Tube
N2A0	150	80	1.5	1.0	0	2	5.57	Composite Tube
N3A64	150	80	1.5	1.5	64	3	5.60	Composite Tube
N3A30	150	80	1.5	1.5	30	3	5.60	Composite Tube
N3A0	150	80	1.5	1.5	0	3	5.60	Composite Tube
N4A64	150	80	1.5	2.0	64	4	5.64	Composite Tube
N4A30	150	80	1.5	2.0	30	4	5.64	Composite Tube
N4A0	150	80	1.5	2.0	0	4	5.64	Composite Tube
AL	150	76	3.0	-	-	-	5.81	Aluminum Alloy Tube

**Table 11:** Ultimate Load Capacity of Short Tube Specimens in Axial Compression Tests.

Specimen Number	*L*/mm	*D*/mm	*t*_a_/mm	*t*_c_/mm	*θ*/°	*n*	λ	*P*_u,1_ /kN	*P*_u,2_ /kN	*P*_u,3_ /kN	*P*_u,avg_ /kN	*η*_s/%_
N2A64	150	80	1.5	1.0	64	2	5.57	215.66	218.26	227.28	220.40	11.26
N2A30	150	80	1.5	1.0	30	2	5.57	228.13	226.8	235.22	230.05	16.14
N2A0	150	80	1.5	1.0	0	2	5.57	234.68	237.97	232.53	235.06	18.67
N3A64	150	80	1.5	1.5	64	3	5.60	219.69	234.89	213.53	222.70	12.43
N3A30	150	80	1.5	1.5	30	3	5.60	247.18	251.07	239.52	245.92	24.15
N3A0	150	80	1.5	1.5	0	3	5.60	250.23	252.58	251.17	251.33	26.88
N4A64	150	80	1.5	2.0	64	4	5.64	226.7	232.03	234.94	231.22	16.73
N4A30	150	80	1.5	2.0	30	4	5.64	256.84	242.58	257.24	252.22	27.33
N4A0	150	80	1.5	2.0	0	4	5.64	262.98	260.75	254.12	259.28	30.89
AL	150	76	3.0	-	-	-	5.81	197.59	198.21	198.46	198.09	-

Note: *L*, *D*, *t*_a_, and *t*_c_ refer to Figure 13.

**Table 12:** Components with Different Initial Imperfections.

Specimen Number	*L*/mm	*D*/mm	*t*_a_/mm	*t*_c_/mm	*θ*/°	*n*	Initial Imperfection	Ultimate Load/kN
Imp-0	150	80	1.5	1.5	64	3	0	244.97
Imp-*D*/1000	150	80	1.5	1.5	64	3	*D*/1000	236.45
Imp-*D*/500	150	80	1.5	1.5	64	3	*D*/500	232.32
Imp-*D*/200	150	80	1.5	1.5	64	3	*D*/200	227.44
Imp-*D*/100	150	80	1.5	1.5	64	3	*D*/100	205.74

**Table 13:** Specimens of Different Sizes.

Specimen Number	*L*/mm	*D*/mm	*t*_a_/mm	*t*_c_/mm	*θ*/°	Ultimate Load /kN
Ta-0.5	150	80	0.5	1.5	60	91.77
Ta-1.0	150	80	1.0	1.5	60	156.96
Ta-1.5	150	80	1.5	1.5	60	222.41
Ta-2.0	150	80	2.0	1.5	60	283.95
Ta-2.5	150	80	2.5	1.5	60	347.15
Tc-0.5	150	80	1.5	0.5	60	207.31
Tc-1.0	150	80	1.5	1.0	60	216.81
Tc-1.5	150	80	1.5	1.5	60	222.41
Tc-2.0	150	80	1.5	2.0	60	226.47
Tc-2.5	150	80	1.5	2.5	60	232.89
D-60	150	60	1.5	1.5	60	164.26
D-70	150	70	1.5	1.5	60	192.25
D-80	150	80	1.5	1.5	60	222.41
D-90	150	90	1.5	1.5	60	247.67
D-100	150	100	1.5	1.5	60	277.80
L-100	100	80	1.5	1.5	60	219.85
L-150	150	80	1.5	1.5	60	222.41
L-200	200	80	1.5	1.5	60	219.99
L-250	250	80	1.5	1.5	60	220.30
L-300	300	80	1.5	1.5	60	222.28
L-350	350	80	1.5	1.5	60	222.23

**Table 14:** Components with Different Fiber Winding Angles.

Specimen Number	*L*/mm	*D*/mm	*t*_a_/mm	*t*_c_/mm	*θ*/°	Ultimate Load /kN
*θ*-0	150	80	1.5	1.5	0	307.72
*θ*-15	150	80	1.5	1.5	15	292.58
*θ*-30	150	80	1.5	1.5	30	277.41
*θ*-45	150	80	1.5	1.5	45	240.19
*θ*-60	150	80	1.5	1.5	60	217.07
*θ*-75	150	80	1.5	1.5	75	233.76
*θ*-90	150	80	1.5	1.5	90	240.92

**Table 15:** Parameters of specimens with different fiber volume ratios.

Specimen Number	*L*/mm	*D*/mm	*t*_a_/mm	*t*_c_/mm	*θ*/°	*n*	*η*	Specimen Type	Ultimate Load/kN
KF-1	150	80	3.0	-	-	-	0.00%	Aluminum Alloy Tube	425.67
KF-2	150	80	2.75	0.5	30	1	8.33%	Composite Tube	418.93
KF-3	150	80	2.5	1.0	30	2	16.67%	Composite Tube	417.09
KF-4	150	80	2.25	1.5	30	3	25.00%	Composite Tube	446.34
KF-5	150	80	2.0	2.0	30	4	33.33%	Composite Tube	457.83
KF-6	150	80	1.75	2.5	30	5	41.67%	Composite Tube	480.51
KF-7	150	80	1.5	3.0	30	6	50.00%	Composite Tube	470.43
KF-8	150	80	1.25	3.5	30	7	58.33%	Composite Tube	446.88
KF-9	150	80	1.0	4.0	30	8	66.67%	Composite Tube	430.12
KF-10	150	80	0.75	4.5	30	9	75.00%	Composite Tube	380.55
KF-11	150	80	0.5	5.0	30	10	83.33%	Composite Tube	335.66
KF-12	150	80	0.25	5.5	30	11	91.67%	Composite Tube	286.17
KF-13	150	80	-	6.0	30	12	100.00%	CFRP Tube	232.46

**Table 16:** Comparison of Calculated Load-Bearing Capacity and Experimental Results for the Axial Compression of Short Tube Components.

Specimen Number	Experimental Average *P*_u,avg_/kN	Numerical Simulation Results *P*_u,n_/kN	Calculated Value *P*_u_/kN	Error with Experimental Value *η*_s,avg_	Error with Numerical Result *η*_s,n_
N2A64	220.40	227.65	222.39	0.90%	−2.31%
N2A30	230.05	236.32	234.45	1.91%	−0.79%
N2A0	235.06	243.81	244.50	4.02%	0.28%
N3A64	222.70	231.00	228.93	2.80%	−0.90%
N3A30	245.92	252.01	246.90	0.40%	−2.03%
N3A0	251.33	278.52	276.84	10.15%	−0.60%
N4A64	231.22	232.50	235.36	1.79%	1.23%
N4A30	252.22	267.92	259.16	2.75%	−3.27%
N4A0	259.28	308.24	304.68	17.51%	−1.15%
AL	198.09	198.29	198.14	0.03%	−0.08%

## Data Availability

The original contributions presented in this study are included in the article. Further inquiries can be directed to the corresponding author.
